# *Rubus chingii* Hu: A Review of the Phytochemistry and Pharmacology

**DOI:** 10.3389/fphar.2019.00799

**Published:** 2019-07-16

**Authors:** Guohua Yu, Zhiqiang Luo, Wubin Wang, Yihao Li, Yating Zhou, Yuanyuan Shi

**Affiliations:** ^1^Shenzhen Hospital, Beijing University of Chinese Medicine, Shenzhen, China; ^2^School of Life Sciences, Beijing University of Chinese Medicine, Beijing, China

**Keywords:** Rubus chingii Hu, phytochemistry, pharmacology, toxicity, quality control

## Abstract

*Rubus chingii* Hu (*R. chingii*), referred to as “Fu-Pen-Zi” in Chinese, has great medicinal and dietary values since ancient times. The dried fruits of *R. chingii* have been widely used in traditional Chinese medicine (TCM) for the treatment of kidney enuresis and urinary frequency for centuries. According to current findings, *R. chingii* has been reported to contain a variety of chemical constituents, mostly triterpenoids, diterpenoids, flavonoids, and organic acids. These compounds have been demonstrated to be the major bioactive components responsible for pharmacological effects such as anticomplementary, anticancer, antioxidant, antimicrobial, and anti-inflammatory functions. Therefore, this review focused on the up-to-date published data of the literature about *R. chingii* and comprehensively summarized its phytochemistry, pharmacology, quality control, and toxicity to provide a beneficial support to its further investigations and applications in medicines and foods.

## Introduction

The genus *Rubus*, belonging to the *Rosaceae* family, has edible and economically important fruits and is widely distributed throughout the Northern Hemisphere (Moreno-Medina et al., 2018). This genus consists of over 700 species, about 194 of which occur in China, including *R. chingii*, *R. idaeus*, *R. rosifolius*, *R. parvifolius*, and so on (Li et al., 2015). Among them, *R. chingii* is an important functional food with the fruits known as “Fu-Pen-Zi” in Chinese. It is mainly cultivated in East China, especially in Jiangxi province, Anhui province, Jiangsu province, Zhejiang province, and Fujian province. Due to its rich nutritional and medicinal value, *R. chingii* has been frequently used in traditional Chinese medicine (TCM) for centuries (Liu and Niu, 2014). The medical properties of *R. chingii* have been mentioned in many landmark Chinese medical monographs, such as “Compendium of Materia Medica,” “Bencao Mengquan,” “Leigong Paozhi Lun,” and “Qianjin Yi Fang.” According to the theory of traditional Chinese herbal medical science, *R. chingii* is commonly used as a tonic for the treatment of enuresis, kidney deficiency, impotence and prospermia, frequency of micturition, spermatorrhea, and other illnesses (Xie et al., 2013a).

Since the universal uses of *R. chingii* in folk medicines, a great deal of studies concerning the chemical constituents and pharmacological activities of this medicinal plant have been carried out, which gave rise to numerous interesting and attractive results. Many *in vitro* and *in vivo* investigations have indicated that the extracts and the ingredients isolated from *R. chingii* possess abundant pharmacological effects, such as anticomplementary, anticancer, antioxidant, antimicrobial, anti-aging and anti-inflammatory activities (Shi, 2017). These marvelous biological functions of this herb can be attributed to the presence of a broad spectrum of phytochemical constituents including triterpenoids, diterpenoids, flavonoids, organic acids, and many other compounds.

Although some brief reviews about the chemical constituents and biological activities have been conducted, these papers were written in Chinese and not studied in a systematic manner. This paper strives for a comprehensive overview of the latest information on the phytochemistry, biological activities, quality control, as well as the toxicity of this herb. More importantly, the correlation between the biological properties and the existence of the bioactive chemical components responsible for the actions has also been discussed based on the published literatures. Finally, the major achievements and shortcomings, together with the possible tendency and perspective for future food and pharmacological research of this herb, have been put forward, too. We believe that this review will highlight the significance of *R. chingii* and indicate new research directions of this species.

## Phytochemical Constituents of *R. chingii*

So far, more than 235 chemical constituents have been isolated and identified from *R. chingii* (**Table 1**). These compounds include 15 triterpenoids, 15 diterpenoids, 18 flavonoids, 7 alkaloids, 95 volatile compounds, 5 coumarins, 9 steroids, 56 organic acids, and 15 other compounds. Among them, triterpenoids and diterpenoids have been identified as the characteristic components.

**Table 1:** Chemical constituents of *R. chingii*.

No.	Chemical component	Part	Molecular formula	References
**TRITERPENOIDS**				
1	Fupenzic acid	Fruit	C_30_H_44_O_5_	Hattori et al., 1988
2	Oleanic acid	Fruit	C_30_H_48_O_3_	Guo, 2005
3	Maslinic acid	Fruit	C_30_H_48_O_4_	Guo, 2005
4	Arjunic acid	Fruit	C_30_H_48_O_5_	Guo, 2005
5	2α, 3α, 19α-trihydroxyolean-12-ene-28-oic-acid	Fruit	C_30_H_48_O_5_	Guo, 2005
6	Sericic acid	Fruit	C_30_H_48_O_6_	Guo, 2005
7	Ursolic acid	Fruit, Root	C_30_H_48_O_3_	Guo, 2005; Cheng, 2008
8	2α-hydroxyursolic acid	Fruit	C_30_H_48_O_4_	Guo, 2005
9	Euscaphic acid	Fruit, Root	C_30_H_48_O_5_	Guo, 2005; Cheng, 2008
10	Hyptatic acid	Fruit	C_30_H_48_O_6_	Guo, 2005
11	11α-hydroxyeuscaphic acid	Root	C_30_H_48_O_6_	Cheng, 2008
12	2α,19α,24-trihydroxyurs-12-ene-3-oxo-28-acid	Fruit	C_30_H_46_O_6_	Chai, 2008
13	Tormentic acid	Fruit	C_30_H_48_O_5_	Chai, 2008
14	Nigaichigoside F1	Fruit	C_36_H_58_O_11_	Xiao et al., 2011
15	2α,19α-dihydroxy-3-oxo-12-ursen-28-oic acid	Fruit	C_30_H_46_O_5_	Xiao et al., 2011
**DITERPENOIDS**				
16	Rubusoside	Leaf	C_32_H_50_O_13_	Tanaka et al., 1981
17	Goshonoside-F1	Leaf	C_26_H_44_O_9_	Tanaka et al., 1981
18	Goshonoside-F2	Leaf	C_27_H_46_O_8_	Tanaka et al., 1981
19	Goshonoside-F3	Leaf	C_32_H_52_O_14_	Tanaka et al., 1981
20	Goshonoside-F4	Leaf	C_32_H_54_O_13_	Tanaka et al., 1981
21	Goshonoside-F5	Leaf	C_32_H_54_O_14_	Tanaka et al., 1981
22	Goshonoside-F6	Leaf, Fruit	C_31_H_52_O_12_	Wang, 1991
23	Goshonoside-F7	Leaf, Fruit	C_32_H_54_O_12_	Wang, 1991
24	Goshonoside-G	Fruit	C_37_H_62_O_17_	Sun et al., 2013b
25	*ent*-Labda-8(17),13*E*-diene-3β,15,18-triol	Fruit	C_20_H_34_O_3_	Guo, 2015
26	*ent*-Labda-8(17),13*E*-diene-3α,15,18-triol	Fruit	C_20_H_34_O_3_	Guo, 2015
27	15,18-Di-*O*-β-D-glucopyranosyl-13(*E *)-*ent*-labda-7(8),13(14)-diene-3β,15,18–triol	Fruit	C_32_H_54_O_13_	Guo, 2015
28	15,18-Di-*O*-β-D-glucopyranosyl-13(*E *)-*ent*-labda-8(9),13(14)-diene-3β,15,18–triol	Fruit	C_32_H_54_O_13_	Guo, 2015
29	15-*O*-β-D-apiofuranosyl-(1→2)β-D-glucopranosyl-18-*O*-β-D-glucopyranosyl-13(*E *)-*ent*-labda-8(9),13(14)-diene-3β,15,18-triol	Fruit	C_37_H_62_O_17_	Guo, 2015
30	*ent*-16α,17-dihydroxy-kauran-19-oic acid	Fruit	C_20_H_32_O_4_	Zhang et al., 2017b
FLAVONOIDS				
31	Kaempferol	Fruit	C_15_H_10_O_6_	Guo, 2005
32	Quercetin	Fruit	C_15_H_10_O_7_	Guo, 2005
33	Tiliroside	Fruit	C_30_H_26_O_13_	Guo, 2005
34	Astragalin	Fruit	C_21_H_20_O_11_	Guo, 2005
35	Quercetin-3-*O*-β-D-glucopyranoside	Fruit	C_21_H_20_O_12_	Guo, 2005
36	Kaempferol-3-*O*-β-D-glucuronic acid methyl ester	Fruit	C_22_H_20_O_12_	Guo, 2005
37	Kaempferol-7-*O*-α-L-rhamnoside	Fruit	C_21_H_20_O_10_	Liu, 2005
38	2”-*O*-Galloyl-hyperin	Fruit	C_28_H_24_O_16_	Liu, 2005
39	Aromadedrin	Fruit	C_15_H_12_O_6_	Cheng, 2008
40	Quercitrin	Fruit	C_21_H_20_O_11_	Cheng, 2008
41	Hyperoside	Fruit	C_21_H_20_O_12_	Cheng, 2008
42	*cis*-Tiliroside	Fruit	C_30_H_26_O_13_	Cheng, 2008
43	Phloridzin	Fruit	C_21_H_24_O_10_	Xiao et al., 2011
44	Kaempferol-3-*O*-hexoside	Fruit	C_21_H_20_O_11_	He et al., 2013
45	Quercetin-3-*O*-glucuronide	Fruit	C_21_H_18_O_13_	He et al., 2013
46	Kaempferol-3-glucuronide	Fruit	C_21_H_18_O_12_	He et al., 2013
47	Kaempferol-3-*O*-β-D-rutinoside	Fruit	C_27_H_30_O_15_	He et al., 2013
48	Rutin	Fruit	C_27_H_30_O_16_	Zhang et al., 2017a
**ALKALOIDS**				
49	4-Hydroxy-2-oxo-1,2,3,4-terahydroquinoline-4-carboxylic acid	Fruit	C_10_H_9_NO_4_	Chai, 2008
50	Methyl 1-oxo-1,2-dihydroisoquinoline-4-carboxylate	Fruit	C_11_H_9_NO_3_	Chai, 2008
51	1-oxo-1,2-Dihydroisoquinoline-4-carboxylic acid	Fruit	C_10_H_7_NO_3_	Chai, 2008
52	Rubusine	Fruit	C_10_H_7_NO_3_	Ding, 2011
53	Methyl (3-hydroxy-2-oxo-2,3-dihydroindol-3-yl)-acetate	Fruit	C_11_H_11_NO_4_	Ding, 2011
54	Methyldioxindole-3-acetate	Fruit	C_11_H_11_NO_4_	Ding, 2011
55	2-oxo-1,2-Dihydroquinoline-4-carboxylic acid	Fruit	C_10_H_7_NO_3_	Ding, 2011
**VOLATILE CONSTITUENTS**				
56	Vitamin E	Fruit	C_29_H_50_O_2_	Zhang and Jiang, 2015
57	2,2,4-Trimethyl-pentane	Leaf, Fruit	C_18_H_18_	Zhang and Jiang, 2015; Han et al., 2014
58	2,2,3,3-Tetramethyl-butane	Leaf	C_18_H_18_	Han et al., 2014
59	1-Hydroxy-2-methyl-1-phenyl-3-pentanone	Leaf	C_12_H_16_O_2_	Han et al., 2014
60	Linalyl acetate	Leaf, Fruit	C_12_H_20_O_2_	Zhang and Jiang, 2015; Han et al., 2014
61	α-Terpinene	Leaf	C_10_H_16_	Han et al., 2014
62	α-Thujene	Leaf	C_10_H_16_	Han et al., 2014
63	2-Ethylhexyl acrylate	Leaf	C_11_H_20_O_2_	Han et al., 2014
64	*trans*-Linalool oxide	Leaf, Fruit	C_10_H_18_O_2_	Zhang and Jiang, 2015; Han et al., 2014
65	*cis*-Linalool oxide	Leaf, Fruit	C_10_H_18_O_2_	Zhang and Jiang, 2015; Han et al., 2014
66	L-α-Terpineol	Leaf	C_10_H_18_O	Han et al., 2014
67	Neryl acetate	Leaf	C_12_H_20_O_2_	Han et al., 2014
68	*cis*-*p*-2-Menthen-1-ol	Leaf	C_10_H_18_O	Han et al., 2014
69	2-(2-Butoxyethoxy)-Ethanol acetate	Leaf	C_12_H_22_O_6_	Han et al., 2014
70	*n*-Tridecane	Leaf	C_13_H_28_	Han et al., 2014
71	5-Oxoheptanoate methyl	Leaf	C_8_H_14_O_3_	Han et al., 2014
72	1-(4-Hydroxymethylphenyl)ethanone	Leaf	C_9_H_10_O_2_	Han et al., 2014
73	Terpineol-4	Leaf, Fruit	C_10_H_18_O	Zhang and Jiang, 2015; Han et al., 2014
74	(*E *)-1-(2,6,6-Trimethyl-1,3-cyclohexadien-1-yl)-2-buten-1-one	Leaf	C_13_H_18_O	Han et al., 2014
75	*trans*-Caryophyl-lene	Leaf	C_15_H_24_	Han et al., 2014
76	Calarene	Leaf, Fruit	C_15_H_24_	Zhang and Jiang, 2015; Han et al., 2014
77	Coniferyl alcohol	Leaf	C_10_H_12_O_3_	Han et al., 2014
78	1-(4,7,7-Trimethyl-3-bicyclo[4.1.0]hept-4-enyl)ethanone	Leaf	C_12_H_18_O	Han et al., 2014
79	*trans*-Dihydrocarvyl acetate	Leaf	C_12_H_20_O_2_	Han et al., 2014
80	*E*-10-Pentadecenol	Leaf	C_15_H_30_O	Han et al., 2014
81	Dodecyl aldehyde	Leaf	C_12_H_24_O	Han et al., 2014
82	12-Methyltridecanal	Leaf	C_14_H_28_O	Han et al., 2014
83	3-Methyloctanedioic acid-dimethyl ester	Leaf	C_11_H_20_O_4_	Han et al., 2014
84	Diisobutyl phthalate	Leaf	C_16_H_22_O_4_	Han et al., 2014
85	Cedryl formate	Leaf	C_16_H_26_O_2_	Han et al., 2014
86	Phytol	Leaf	C_20_H_40_O	Han et al., 2014
87	3-Methyl-2-pentanone	Fruit	C_6_H_12_O	Pi and Wu, 2003
88	2-Methoxyethyl acetate	Fruit	C_5_H_10_O_3_	Pi and Wu, 2003
89	3-Methyl-2-pentane	Fruit	C_7_H_10_N_2_O	Pi and Wu, 2003
90	1,1-diethoxyethane	Fruit	C_6_H_14_O_2_	Pi and Wu, 2003
91	2,5-Dimethylfuran	Fruit	C_6_H_8_O	Pi and Wu, 2003
92	2-Hexanal	Fruit	C_6_H_12_O	Pi and Wu, 2003
93	Xylene	Fruit	C_8_H_10_	Pi and Wu, 2003
94	Ethylbenzene	Fruit	C_8_H_10_	Pi and Wu, 2003
95	Ethyl formate	Fruit	C_3_H_6_O_2_	Pi and Wu, 2003
96	2-Butanone	Fruit	C_4_H_8_O	Pi and Wu, 2003
97	Isovaleraldehyde	Fruit	C_5_H_10_O	Pi and Wu, 2003
98	Ethyl acetate	Fruit	C_4_H_8_O_2_	Pi and Wu, 2003
99	2-Methylpentane	Fruit	C_6_H_14_	Pi and Wu, 2003
100	2-Heptanol	Fruit	C_7_H_16_O	Pi and Wu, 2003
101	Hexaldehyde	Fruit	C_6_H_12_O	Pi and Wu, 2003
102	1-Hexene	Fruit	C_6_H_12_	Pi and Wu, 2003
103	1-Methyl-3-isopropylbenzene	Fruit	C_10_H_14_	Dian et al., 2005
104	1,2,3,5-Tetramethylbenzene	Fruit	C_10_H_14_	Dian et al., 2005
105	Durene	Fruit	C_10_H_14_	Dian et al., 2005
106	3-Ethylstyrene	Fruit	C_10_H_12_	Dian et al., 2005
107	2,4-Dimethylstyrene	Fruit	C_10_H_12_	Dian et al., 2005
108	2,6-Dimethylcyclohexanol	Fruit	C_8_H_16_O	Dian et al., 2005
109	1-Hexadecanol	Fruit	C_16_H_34_O	Dian et al., 2005
110	Hexahydrofarnesyl acetone	Fruit	C_18_H_36_O	Dian et al., 2005
111	*n*-Hexadecanal	Fruit	C_16_H_32_O	Dian et al., 2005
112	14-Methyl-pentadecanoic acid, methyl ester	Fruit	C_17_H_34_O_2_	Dian et al., 2005
113	Ambrettolide	Fruit	C_16_H_28_O_2_	Dian et al., 2005
114	Nonadecane	Fruit	C_19_H_40_	Zhang and Jiang, 2015
115	2-Methylnonadecane	Fruit	C_20_H_42_	Zhang and Jiang, 2015
116	Eicosane	Fruit	C_20_H_42_	Zhang and Jiang, 2015
117	α-Pinene	Fruit	C_10_H_16_	Zhang and Jiang, 2015
118	Bicyclo[3.1.0]hexane, 4-methylene-1-(1-methylethyl)-	Fruit	C_10_H_16_	Zhang and Jiang, 2015
119	Eucalyptol	Fruit	C_10_H_18_O	Zhang and Jiang, 2015
120	*p*-Cymene	Fruit	C_10_H_14_	Zhang and Jiang, 2015
121	*trans*-Sabinene hydrate	Fruit	C_10_H_18_O	Zhang and Jiang, 2015
122	γ-Terpinene	Fruit	C_10_H_16_	Zhang and Jiang, 2015
123	Linalool	Fruit	C_10_H_18_O	Zhang and Jiang, 2015
124	β-*trans*-Ocimene	Fruit	C_10_H_16_	Zhang and Jiang, 2015
125	Methyl thymyl ether	Fruit	C_11_H_16_O	Zhang and Jiang, 2015
126	β-Elemene	Fruit	C_15_H_24_	Zhang and Jiang, 2015
127	α-Cedrene	Fruit	C_15_H_24_	Zhang and Jiang, 2015
128	4,7,9-Megastigmatrien-3-one	Fruit	C_13_H_18_O	Zhang and Jiang, 2015
129	Tridecanoic acid, methyl ester	Fruit	C_14_H_28_O_2_	Zhang and Jiang, 2015
130	Linolenyl alcohol	Fruit	C_18_H_32_O	Zhang and Jiang, 2015
131	Hexadecanoic acid, ethyl ester	Fruit	C_18_H_36_O_2_	Zhang and Jiang, 2015
132	9,12,15-Octadecatrienal	Fruit	C_18_H_30_O	Zhang and Jiang, 2015
133	9,12-Octadecadienoic acid, methyl ester	Fruit	C_19_H_34_O_2_	Zhang and Jiang, 2015
134	Octadecane, 2-methyl-	Fruit	C_19_H_40_	Zhang and Jiang, 2015
135	(9*Z*,12 *Z*)-Methyl octadeca-9,12-dienoate	Fruit	C_19_H_34_O_2_	Zhang and Jiang, 2015
136	Methyl linolenate	Fruit	C_19_H_32_O_2_	Zhang and Jiang, 2015
137	Linoleic acid ethyl ester	Fruit	C_20_H_36_O_2_	Zhang and Jiang, 2015
138	Ethyl linolenate	Fruit	C_20_H_34_O_2_	Zhang and Jiang, 2015
139	(2*E*)-3,7,11,15-Tetramethyl-2-hexadecen-1-ol	Fruit	C_20_H_40_O	Zhang and Jiang, 2015
140	9-Octadecenamide, (*Z*)-	Fruit	C_18_H_35_NO	Zhang and Jiang, 2015
141	Tetracosane	Fruit	C_24_H_50_	Zhang and Jiang, 2015
142	Heptacosane	Fruit	C_27_H_56_	Zhang and Jiang, 2015
143	9,12-Octadecadienoic acid (*Z,Z*)-,2,3-bis [(trimethylsilyl)oxy]propylester	Fruit	C_27_H_54_O_4_Si_2_	Zhang and Jiang, 2015
144	Octacosane	Fruit	C_28_H_58_	Zhang and Jiang, 2015
145	Supraene	Fruit	C_30_H_50_	Zhang and Jiang, 2015
146	Nonacosane	Fruit	C_29_H_60_	Zhang and Jiang, 2015
147	δ-Tocopherol	Fruit	C_27_H_46_O_2_	Zhang and Jiang, 2015
148	β-Tocopherol	Fruit	C_28_H_48_O_2_	Zhang and Jiang, 2015
149	γ-Tocopherol	Fruit	C_28_H_48_O_2_	Zhang and Jiang, 2015
150	Di-*n*-butyl phthalate	Fruit	C_16_H_22_O_4_	Zhang and Jiang, 2015
**COUMARINS**				
151	Esculetin	Fruit	C_9_H_6_O_4_	Liu, 2005
152	Esculin	Fruit	C_15_H_16_O_9_	Liu, 2005
153	Imperatorin	Fruit	C_16_H_14_O_4_	Liu, 2005
154	Rubusin A	Fruit	C_12_H_8_O_6_	Sun et al., 2011
155	Rubusin B	Fruit	C_12_H_6_O_7_	Liang et al., 2015
**STEROIDS**				
156	β-Sitosterol	Fruit, Root	C_29_H_50_O	Guo, 2005; Cheng, 2008
157	Daucosterol	Fruit, Root	C_35_H_60_O_6_	Guo, 2005; Cheng, 2008
158	Stigmast-4-ene-(3β,6α)-diol	Fruit	C_29_H_50_O_2_	Guo, 2005
159	Stigmast-5-en-3-ol,oleate	Fruit	C_47_H_82_O_2_	You, 2009
160	β-Stigmasterol	Fruit	C_29_H_48_O	Xiao, 2011
161	7α-Hydroxy-β-sitosterol	Fruit	C_29_H_50_O_2_	Du et al., 2014
162	Sitosterol palmitate	Fruit	C_45_H_78_O_2_	Liu et al., 2014
163	Campesterol	Fruit	C_28_H_48_O	Zhang and Jiang, 2015
164	γ-Sitosterol	Fruit	C_29_H_50_O	Zhang and Jiang, 2015
**ORGANIC ACIDS**				
***Phenolic acids***				
165	4-Hydroxybenzoic acid	Fruit	C_7_H_6_O_3_	Cheng, 2008
166	Ellagic acid	Fruit	C_14_H_6_O_8_	Cheng, 2008
167	Ethyl gallate	Fruit	C_9_H_10_O_5_	Cheng, 2008
168	5-[3-Hydroxymethyl-5-(3-hydroxypropyl)-7-Methoxyl-2,3-dihydro-benzofuran-2-yl]-2-methoxy-phenol	Fruit	C_20_H_24_O_6_	Guo, 2015
169	4-Hydroxy-3-methoxy benzoic acid	Fruit	C_8_H_8_O_4_	You, 2009
170	Gallic acid	Fruit	C_7_H_6_O_5_	Xie et al., 2005
171	Resveratrol	Fruit	C_14_H_12_O_3_	Lim et al., 2004
172	Methyl brevifolin-carboxylate	Fruit	C_14_H_10_O_8_	Xiao et al., 2011
173	Liballinol	Fruit	C_18_H_18_O_4_	You, 2009
174	4-Hydrobenzaldehyde	Fruit	C_7_H_6_O_2_	You, 2009
175	Vanillic acid	Fruit	C_8_H_8_O_4_	Liu, 2005
176	Raspberry ketone	Fruit	C_10_H_12_O_2_	Zhang, 2014
177	Brevifolin carboxylic acid	Fruit	C_13_H_8_O_8_	Chai et al., 2016
178	4-[3-Hydroxymethyl-5-(3-hydroxypropyl)-2,3-dihydrobenzofuran-2-yl]-2-methoxyphenol	Fruit	C_19_H_22_O_5_	Guo, 2015
179	*p*-Coumaric acid	Fruit	C_9_H_8_O_3_	Li et al., 2018
180	Ellagic acid hexuronide	Fruit	C_20_H_14_O_14_	Li et al., 2018
181	Salicylic acid	Fruit	C_7_H_6_O_3_	Du et al., 2014
182	4-[(2*S*,3*R*)-3-(Hydroxymethyl)-5-(3-hydroxypropyl)-7-methoxy-2,3-dihydro-1-benzofuran-2-yl]-2-methoxyphenol	Fruit	C_20_H_24_O_6_	Chai, 2008
183	Ferulic acid	Fruit	C_10_H_10_O_4_	Liu, 2005
184	4-Hydroxy-3-methoxybenzoic acid	Fruit	C_8_H_8_O_4_	Xie et al., 2005
185	Vanillin	Fruit	C_8_H_8_O_3_	You et al., 2009
186	4-Hydroxyphenylacetic acid	Fruit	C_8_H_8_O_3_	Cheng, 2008
187	Hexacosyl *p*-coumarate	Fruit	C_35_H_60_O_3_	Guo, 2005
Fatty acids				
188	Dotriacontanoic acid	Fruit	C_32_H_64_O_2_	Xie et al., 2005
189	Hexadecanoic acid	Fruit	C_16_H_32_O_2_	Han et al., 2013
190	Stearic acid	Fruit	C_18_H_36_O_2_	Xie et al., 2005
191	Caproic acid	Fruit	C_6_H_12_O_2_	Pi and Wu, 2003
192	*n*-Heptadecanoic acid	Fruit	C_17_H_34_O_2_	Dian et al., 2005
193	Linoleic acid	Fruit	C_18_H_32_O_2_	Zhang and Jiang, 2015
194	2-Hexadecenoic acid	Fruit	C_16_H_30_O_2_	Liu et al., 2014
195	Caprylic acid	Fruit	C_8_H_16_O_2_	Pi and Wu, 2003
196	*n*-Tetracosyl-*p*-coumarate	Fruit	C_33_H_56_O_3_	Du et al., 2014
197	Octadecanoic acid	Fruit	C_18_H_36_O_2_	Zhang and Jiang, 2015
198	9-Octadecynoic acid	Fruit	C_18_H_32_O_2_	Zhang and Jiang, 2015
199	Oleic acid	Fruit	C_18_H_34_O_2_	Dian et al., 2005
200	*N*-pentadecanoic acid	Fruit	C_15_H_30_O_2_	Dian et al., 2005
201	α-Linolenic acid	Leaf, Fruit	C_18_H_30_O_2_	Zhang and Jiang, 2015 Han et al., 2014
202	Tetradecanoic acid	Leaf	C_14_H_28_O_2_	Han et al., 2014
203	Undecanoic acid	Leaf	C_11_H_22_O_2_	Han et al., 2014
204	*trans*-Traumatic acid	Leaf	C_12_H_20_O_4_	Han et al., 2014
205	Dodecanoic acid	Leaf	C_12_H_24_O_2_	Han et al., 2014
206	*n*-Hexacosylferulate	Fruit	C_36_H_62_O_4_	Du et al., 2014
207	8,11,14-Eicosatrienoic acid	Fruit	C_20_H_34_O_2_	Zhang and Jiang, 2015
Tannins				
208	Casuariin	Fruit	C_34_H_24_O_22_	Li et al., 2018
209	Casuarictin	Fruit	C_41_H_28_O_26_	Li et al., 2018
210	Casuarinin	Fruit	C_41_H_28_O_26_	Li et al., 2018
211	Pedunculagin	Fruit	C_34_H_24_O_22_	Li et al., 2018
Others				
212	Oxalic acid	Fruit	C_2_H_2_O_4_	Sun et al., 2013a
213	Tartaric acid	Fruit	C_4_H_6_O_6_	Sun et al., 2013a
214	Acetic acid	Leaf	C_2_H_4_O_2_	Han et al., 2014
215	Malic acid	Fruit	C_4_H_6_O_5_	Sun et al., 2013a
216	Citric acid	Fruit	C_6_H_8_O_7_	Sun et al., 2013a
217	2-Hydroxyquinoline-4-carboxylic acid	Fruit	C_10_H_7_NO_3_	Cheng, 2008
218	Shikimic acid	Fruit	C_7_H_10_O_5_	Liu, 2005
219	Phthalic acid	Fruit	C_8_H_6_O_4_	Zhang and Jiang, 2015
220	Mono-*n*-butyl phthalate	Fruit	C_12_H_14_O_4_	Xie et al., 2013b
**OTHER COMPOUNDS**				
221	Di(2-ethylhexyl) phthalate	Fruit	C_24_H_38_O_4_	Cheng, 2008
222	Ascorbic acid	Fruit	C_8_H_8_O_6_	Sun et al., 2013a
223	Heptadecanoic acid, 14-methyl-, methyl ester	Fruit	C_19_H_38_O_2_	Zhang and Jiang, 2015
224	1-Hexacosanol	Fruit	C_26_H_54_O	You, 2009
225	Adenosine	Fruit	C_10_H_13_N_5_O_4_	Du et al., 2014
226	H-2-indenone,2,4,5,6,7,7α-hexahydro-3-(1-methylethyl)-7α-methyl	Fruit	C_13_H_20_O	You, 2009
227	Butyl dosocanoate	Fruit	C_26_H_52_O_2_	Guo, 2005
228	Uridine	Fruit	C_9_H_12_N_2_O_6_	Kong et al., 2011
229	Methy-β-D-glucopyranoside	Fruit	C_7_H_14_O_6_	Xiao et al., 2011
230	Pentacosanol	Fruit	C_25_H_52_O	Guo, 2005
231	Triacontanol	Fruit	C_30_H_62_O	Chai, 2008
232	Hentriacontane	Fruit	C_31_H_64_	Guo et al., 2007
233	Guanosine	Fruit	C_10_H_13_N_5_O_5_	Kong et al., 2011
234	Glucose	Fruit	C_6_H_12_O_6_	You, 2009
235	3,7-Dihydoxy-1,5-dynitrogen cyclooctane	Fruit	C_6_H_14_N_2_O_2_	Xie et al., 2013b

### Triterpenoids

**Figure 1:**
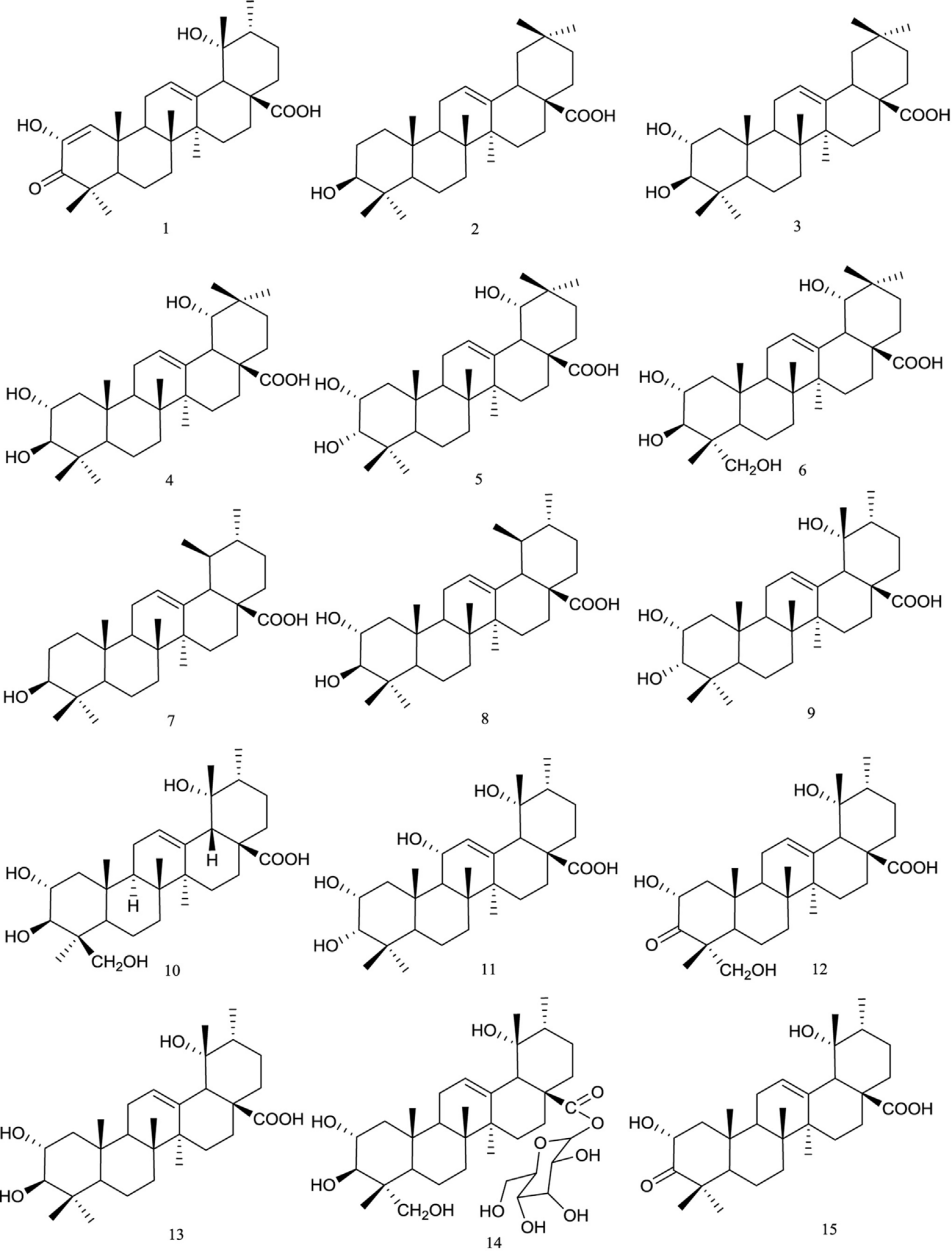
Chemical structures of triterpenoids **(1–15)** isolated from *R. chingii*.

Triterpenoids are the major chemical compounds present in *R. chingii*. They are mainly pentacyclic triterpenoids or thereof derivatives, with oleanane-type and ursane-type skeletons (**Figure 1**). The first study of triterpenes identified in *R. chingii* dates back to the 1980s, when Masao et al. reported the isolation of a new diosphenol-type triterpene named fupenzic acid **(1)** (Hattori et al., 1988). In another work (Guo, 2005), the fruits of *R. chingii* were extracted with methanol. Further fractionation of the methanol extract led to the isolation of five oleanane-type triterpene acids [oleanic acid **(2)**, maslinic acid **(3)**, arjunic acid **(4)**, 2α, 3α, 19α-trihydroxyolean-12-ene-28-oic-acid **(5)**, and sericic acid **(6)**] together with four ursane-type triterpene acids [ursolic acid **(7)**, 2α-hydroxyursolic acid **(8)**, euscaphic acid **(9)**, and hyptatic acid **(10)**]. Moreover, Cheng et al. found that the roots of this plant were rich in triterpenoids. They obtained three triterpene acids, namely, ursolic acid **(7)**, euscaphic acid **(9)**, and 11α-hydroxyeuscaphic acid **(11)** from this plant part (Cheng, 2008). In further studies, Chai et al. obtained 2α,19α,24-trihydroxyurs-12-ene-3-oxo-28-acid **(12)** and tormentic acid **(13)** from the 95% ethanol extract of *R. chingii* fruit (Chai, 2008). Lately, investigation of the 80% ethanol extract of the fruits of *R. chingii* yielded nigaichigoside F1 **(14)** and 2α,19α-dihydroxy-3-oxo-12-ursen-28-oic acid **(15)** (Xiao et al., 2011).

### Diterpenoids

**Figure 2:**
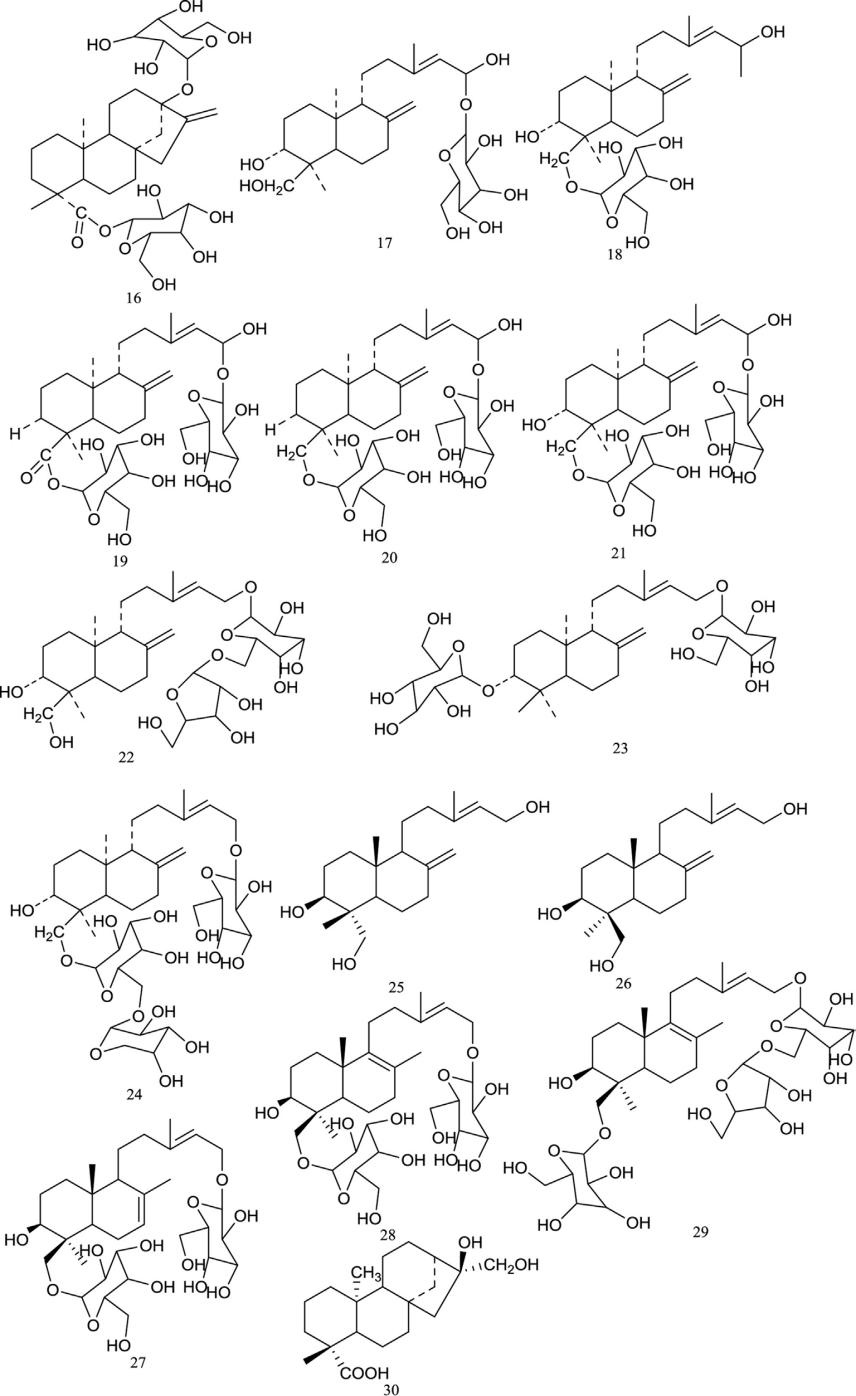
Chemical structures of diterpenoids **(16–30)** isolated from *R. chingii*.

Diterpenoids are also characterized as the representative ingredients of *R. chingii*. Currently, 15 diterpenoids (**Figure 2**), including 2 kaurane-type diterpenoids and 13 labdane-type diterpenoids, have been identified in *R. chingii*. Rubusoside**(16)** was the first diterpenoid isolated from the methanol extract of the leaves of *R. chingii* in 1981 (Tanaka et al., 1981), and subsequent investigations have led to the isolation of five additional labdane-type diterpene glucosides (Goshonoside-F1-F5, **17–21**) (Tanaka et al., 1984). Furthermore, another two labdane-type diterpene glucosides, namely, goshonoside-F6**(22)** and goshonoside-F7**(23)**, were reported to be obtained from both the leaves and fruits of *R. chingii* (Wang, 1991). In 2013, a new ent-labdane diterpene saponin, named goshonoside-G**(24)**, was separated from the 70% ethanol extract of *R. chingii* unripe fruit, and its structure was determined based on NMR spectroscopic studies and mass spectrometry data (Sun et al., 2013b). Later, from the ethyl acetate extract of *R. chingii* fruit, Guo (2015) isolated five labdane-type diterpene glycosides that were elucidated as *ent*-Labda-8(17),13E-diene-3β,15,18-triol**(25)**, *ent*-Labda-8(17),13E-diene-3α,15,18-triol**(26)**, 15,18-di-O-β-D-glucopyranosyl-13(E)-ent-labda-7(8),13(14)-diene-3β,15,18-triol(27), 15,18-di-O-β-D-glucopyranosyl-13(E)-ent-labda-8(9),13(14)-diene-3β,15,18-triol(28), and 15-O-β-D-apiofuranosyl-(1→2)β-D-glucopranosyl-18-O-β-D-glucopyranosyl-13(E)-ent-labda-8(9),13(14)-diene-3β,15,18-triol(29). More recently, Zhang et al. (2017b) found a kaurane-type diterpenoid called ent-16α,17-dihydroxy-kauran-19-oic acid**(30)** from fruits of *R. chingii* by bio-guided isolation.

### Flavonoids

**Figure 3:**
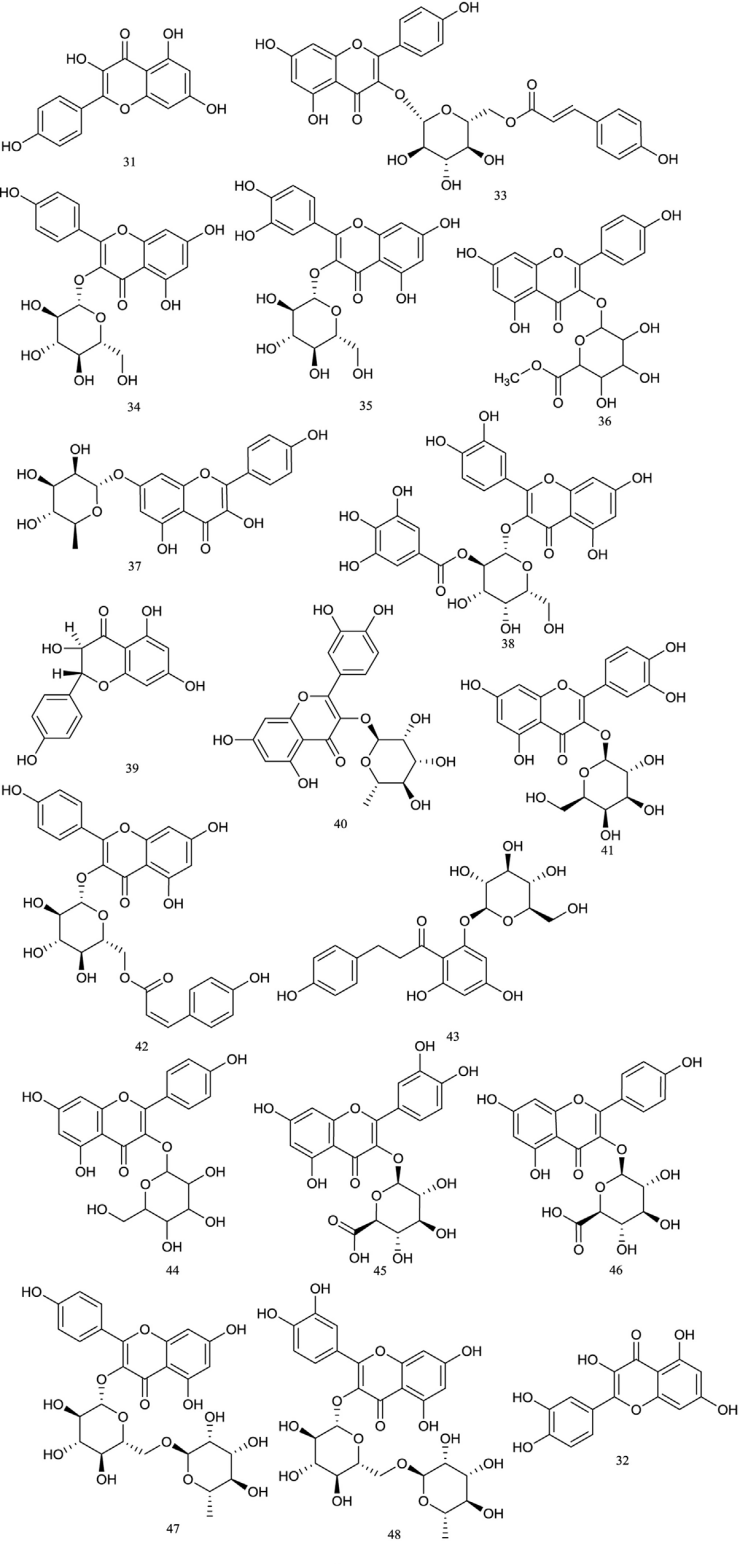
Chemical structures of flavonoids **(31–48)** isolated from *R. chingii*.

Flavonoids, occurring naturally in dietary and medicinal plants (Azietaku et al., 2017), are important polyphenol constituents with various pharmacological effects (Cai et al., 2018). The main types of flavonoids found in *R. chingii* were kaempferol, quercetin, and their derivatives. To date, a total of 18 flavonoids have been reported mainly from the fruits of *R. chingii*. Guo et al. isolated six compounds: kaempferol**(31)**, quercetin**(32)**, tiliroside**(33)**, astragalin**(34)**, quercetin-3-O-β-D-glucopyranoside**(35)**, and kaempferol-3-O-β-D-glucuronic acid methyl ester**(36)** (Guo, 2005). In the same year, Liu (2005) obtained kaempferol-7-O-α-L-rhamnoside**(37)** and 2″-*O*-Galloyl-hyperin**(38)**. Then, by using a series of chromatographic and spectrum technologies, Cheng (2008) isolated and identified aromadedrin**(39)**, quercitrin**(40)**, hyperoside**(41)**, and *cis*-tiliroside**(42)** in 2008. Furthermore, investigation of the 80% ethanol extract of the dried fruits of *R. chingii* yielded phlorizin**(43)** (Xiao et al., 2011). Lately, kaempferol-3-*O*-hexoside**(44)**, quercetin-3-*O*-glucuronide**(45)**, and kaempferol-3-*O*-glucuronide**(46)** were identified in the fruits of *R. chingii* by high-performance liquid chromatography (HPLC) coupled with linear ion trap-OrbiTrap hybrid mass spectrometer (Li et al., 2018). In addition, kaempferol-3-O-β-D-rutinoside**(47)** (He et al., 2013) and rutin**(48)** (Zhang et al., 2017a) were also found in this plant. Their structures are shown in **Figure 3**.

### Alkaloids

**Figure 4:**
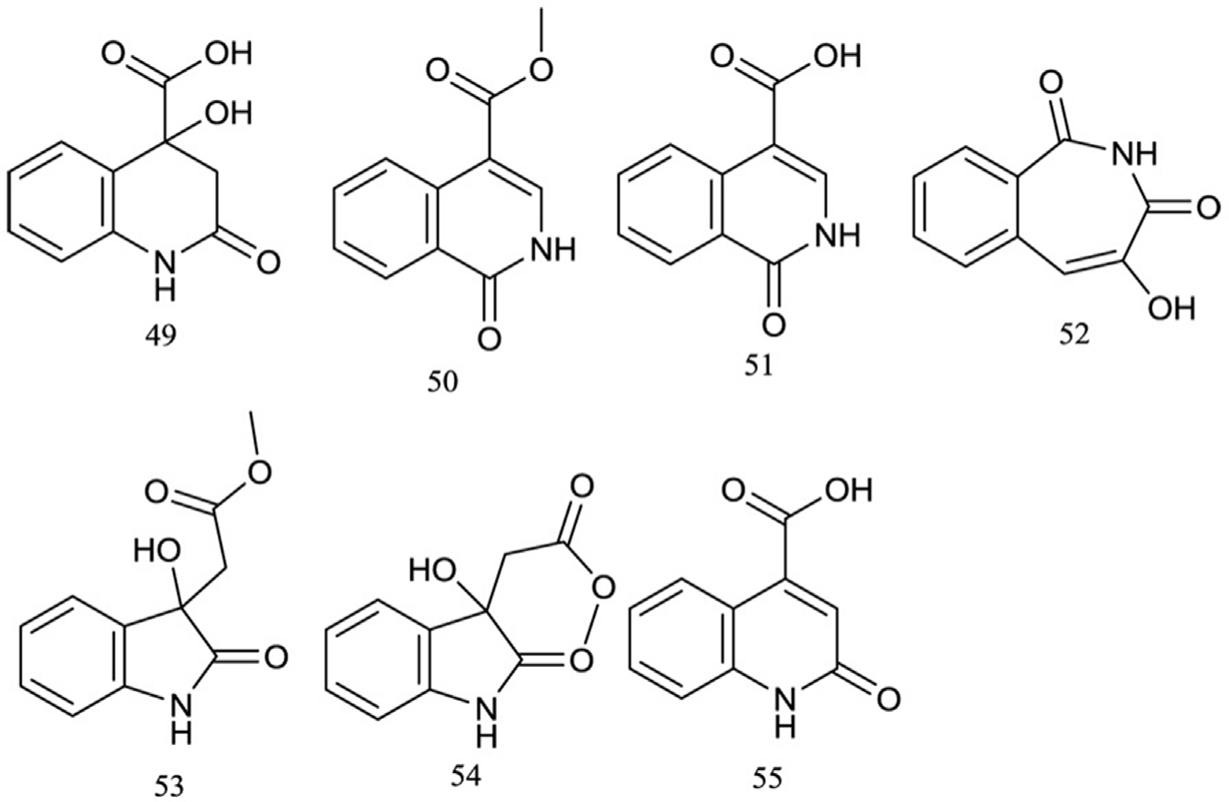
Chemical structures of alkaloids **(49–55)** isolated from *R. chingii*.

Alkaloids represent a relatively small class of compounds in *R. chingii*. Only seven of this class of compounds have been isolated from *R. chingii* (**Figure 4**), with skeletons of the quinoline, isoquinoline, and indole types. In 2008, Chai (2008) reported that from the 95% and 50% ethanol extract of the fruits of *R. chingii*, three alkaloids were isolated and identified as 4-hydroxy-2-oxo-1,2,3,4-terahydroquinoline-4-carboxylic acid**(49)**, methyl 1-oxo-1, 2-dihydroisoquinoline-4-carboxylate**(50)**, and 1-oxo-1, 2-dihydroisoquinoline-4-carboxylic acid**(51)**. In 2011, guiding with 1,1-diphenyl-2-picrylhydrazyl (DPPH) free radical scavenging activity, another four alkaloids, including rubusine**(52)**, methyl (3-hydroxy-2-oxo-2,3-dihydroindol-3-yl)-acetate**(53)**, methyldioxindole-3-acetate**(54)**, and 2-oxo-1,2-dihydroquinoline-4-carboxylic acid**(55)**, were isolated from the ethanol extract of the same plant part (Ding, 2011).

### Volatile Constituents

**Figure 5:**
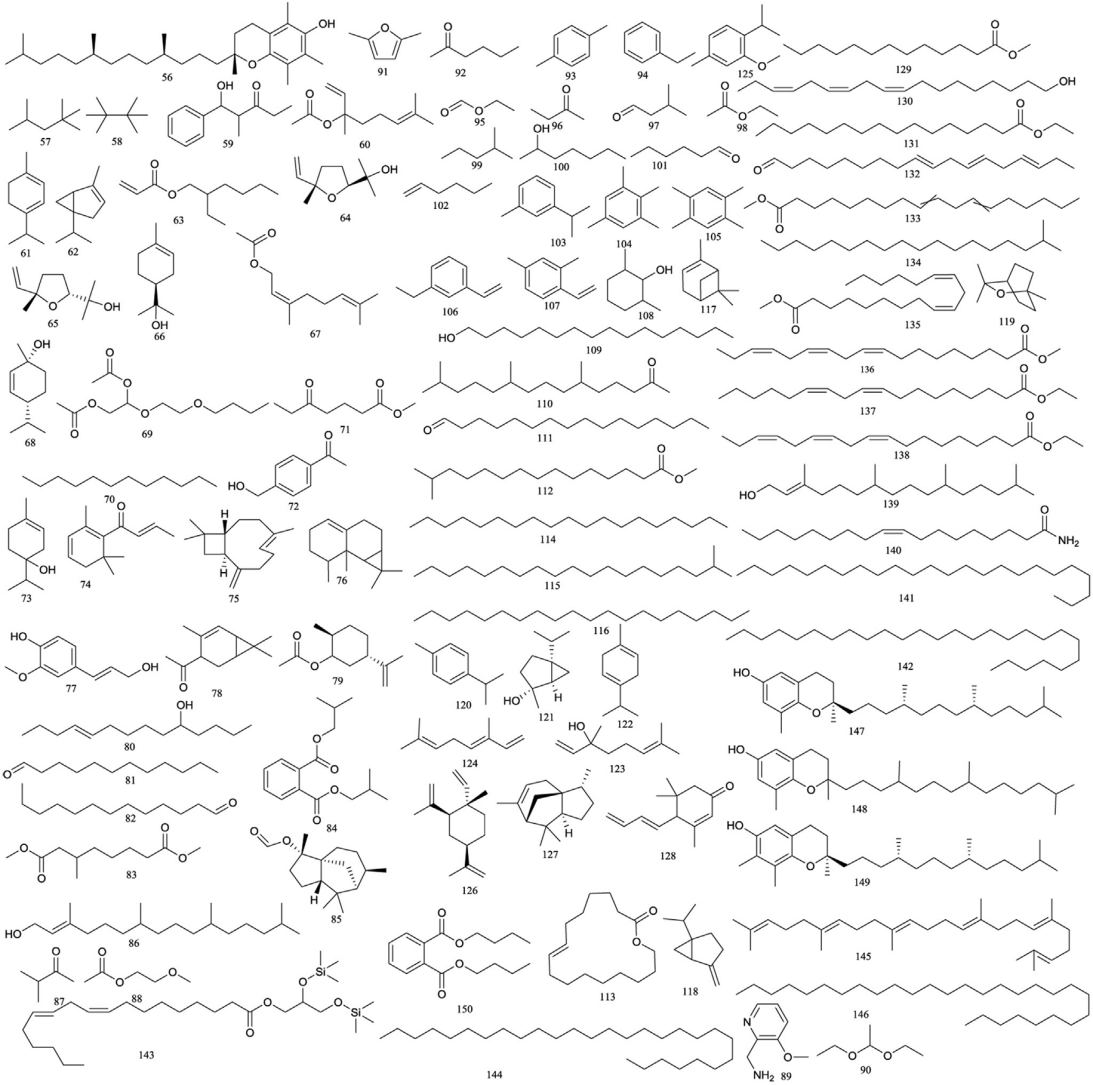
Chemical structures of volatile compounds **(56–150)** isolated from *R. chingii*.

Volatile compounds (**Figure 5**) comprise an important part of *R. chingii* (Pi and Wu, 2003; Dian et al., 2005; Han et al., 2014; Zhang and Jiang, 2015). Han et al. (2014) investigated the volatile constituents from the leaves of *R. chingii* by employing head-space gas chromatography–mass spectrometry (GC/MS) and identified 37 constituents, mainly including hexadecanoic acid (44.97%), tetradecanoic acid (10.88%), and acetic acid (4.13%). In another study conducted in 2015, a total of 58 volatile compounds were identified from the unripe fruits of *R. chingii* using GC/MS (Zhang and Jiang, 2015). According to their structures, these volatile compounds could be divided into eight chemical groups: saturated hydrocarbons (9 compounds), unsaturated hydrocarbons (10 compounds), alcohols (9 compounds), carbonyl compounds (2 compounds), esters (11 compounds), organic acids (7 compounds), oxides and epoxides (8 compounds), and others (2 compounds).

### Coumarins

**Figure 6:**
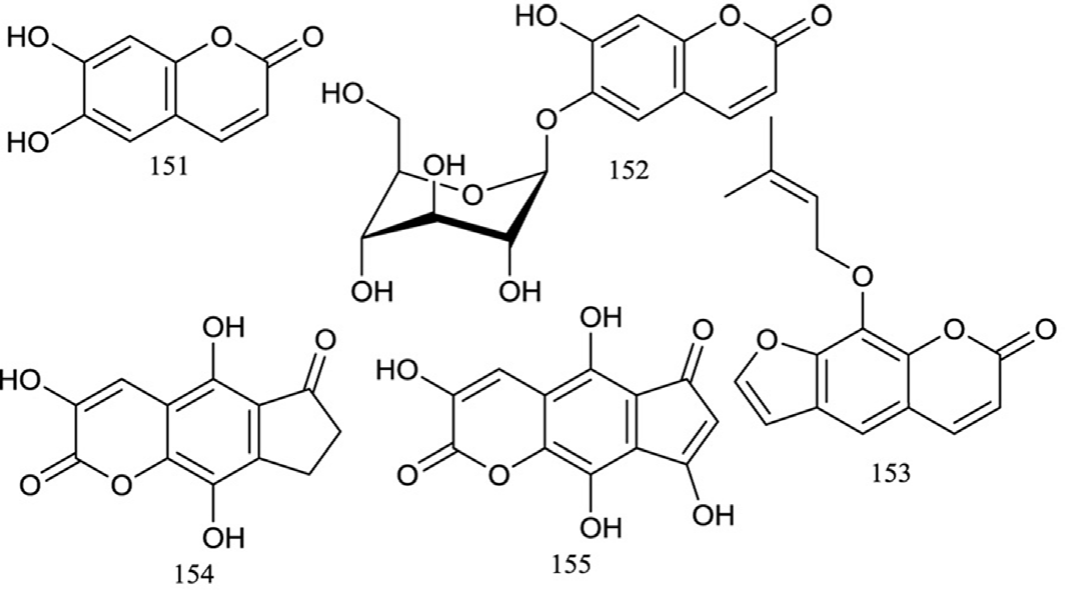
Chemical structures of coumarins **(151–155)** isolated from *R. chingii*.

Coumarins are phenolic compounds characterized by a benzene ring attached to a pyrone ring. They have a fragrant smell and exist throughout the plant kingdom (Azietaku et al., 2017). To date, limited studies have been performed to investigate the coumarins in *R. chingii* and only five coumarins have been isolated, including two simple coumarins and three furocoumarins (**Figure 6**). Liu (2005) isolated and identified esculetin**(151)**, esculin**(152)**, and imperatorin**(153)** from the 70% ethanol extract of the fruits of *R. chingii* by various chromatographic methods. You reported the isolation and structure elucidation of a new furocoumarins, 3,5,9-trihydroxy-7,8-dihydrocyclopenta[g]chromene-2,6-dione**(154)**, which they named Fu-Pen-Zi-Su (You, 2009) or rubusin A (Sun et al., 2011), from the *n*-butanol extract of the fruits of *R. chingii*. Recently, phytochemical analysis of *R. chingii* afforded a new chromone called rubusin B**(155)**, which was confirmed according to the 1D and 2D NMR data and MS data (Liang et al., 2015).

### Steroids

**Figure 7:**
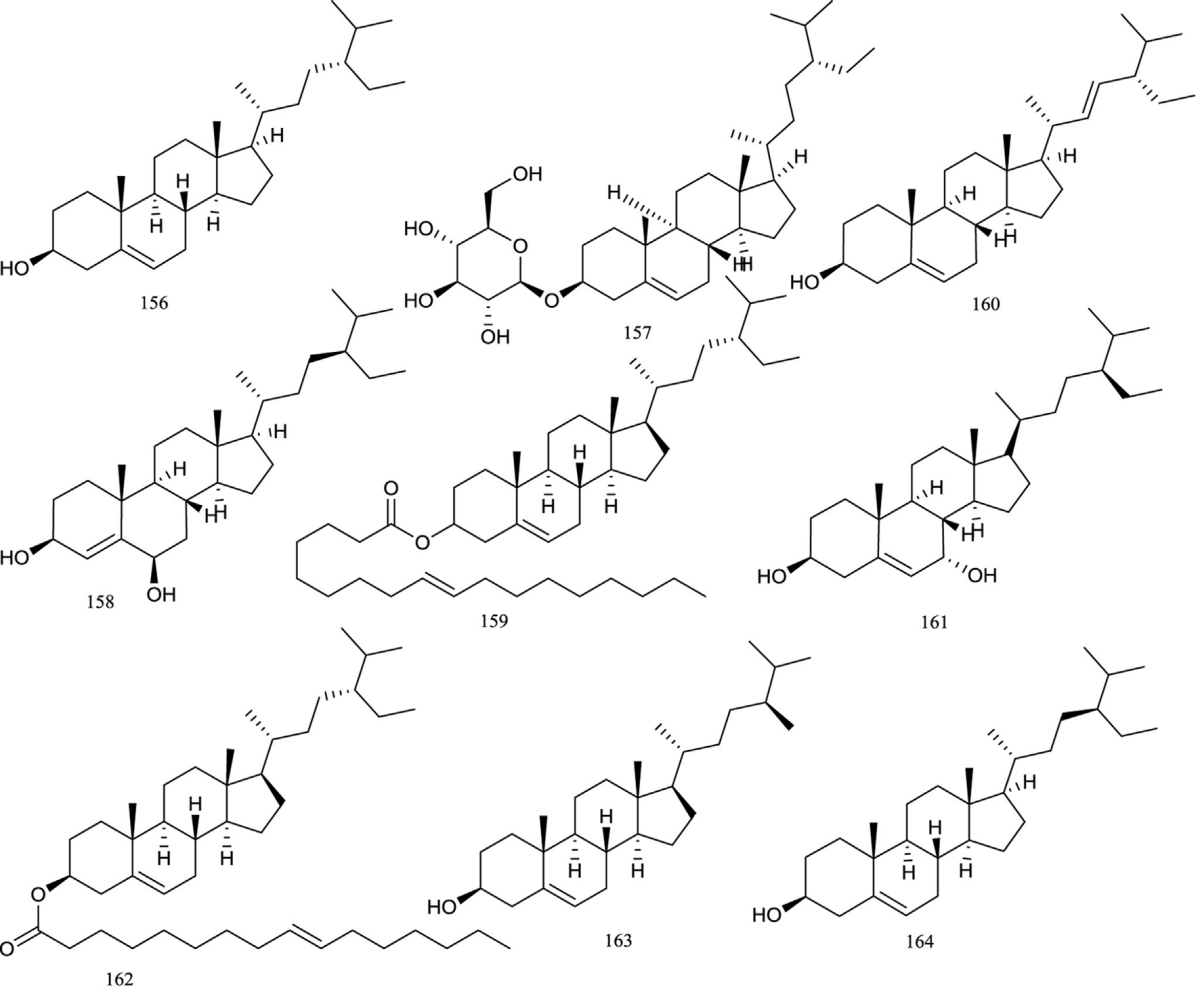
Chemical structures of steroids **(156–154)** isolated from *R. chingii*.

Phytosterols are a class of physiologically active compounds extensively used in cosmetics, foods, and medicines. In *R. chingii*, steroids are relatively rare, and only nine steroidal metabolites have been reported and characterized (**Figure 7**). In 2005, three steroids, namely, β-sitosterol**(156)**, daucosterol**(157)**, and stigmast-4-ene-(3β,6α)-diol**(158)** (Guo, 2005), were found to exist in methanol extract of the fruits of *R. chingii*. Moreover, β-sitosterol**(156)** and daucosterol **(157)** were isolated from the roots of *R. chingii* by Cheng in 2008 (Cheng, 2008). In further studies, another steroid called stigmast-5-en-3-ol,oleate**(159)** was obtained from the methylene chloride extract of *R. chingii* fruit (You, 2009). Other steroidal compounds that were isolated from this plant were β-stigmasterol**(160)** (Xiao, 2011), 7α-hydroxy-β-sitosterol**(161)** (Du et al., 2014), and sitosterol palmitate **(162)** (Liu et al., 2014). In addition, campesterol**(163)** and γ-sitosterol**(164)** were tentatively elucidated by GC/MS (Zhang and Jiang, 2015).

### Organic Acids

**Figure 8:**
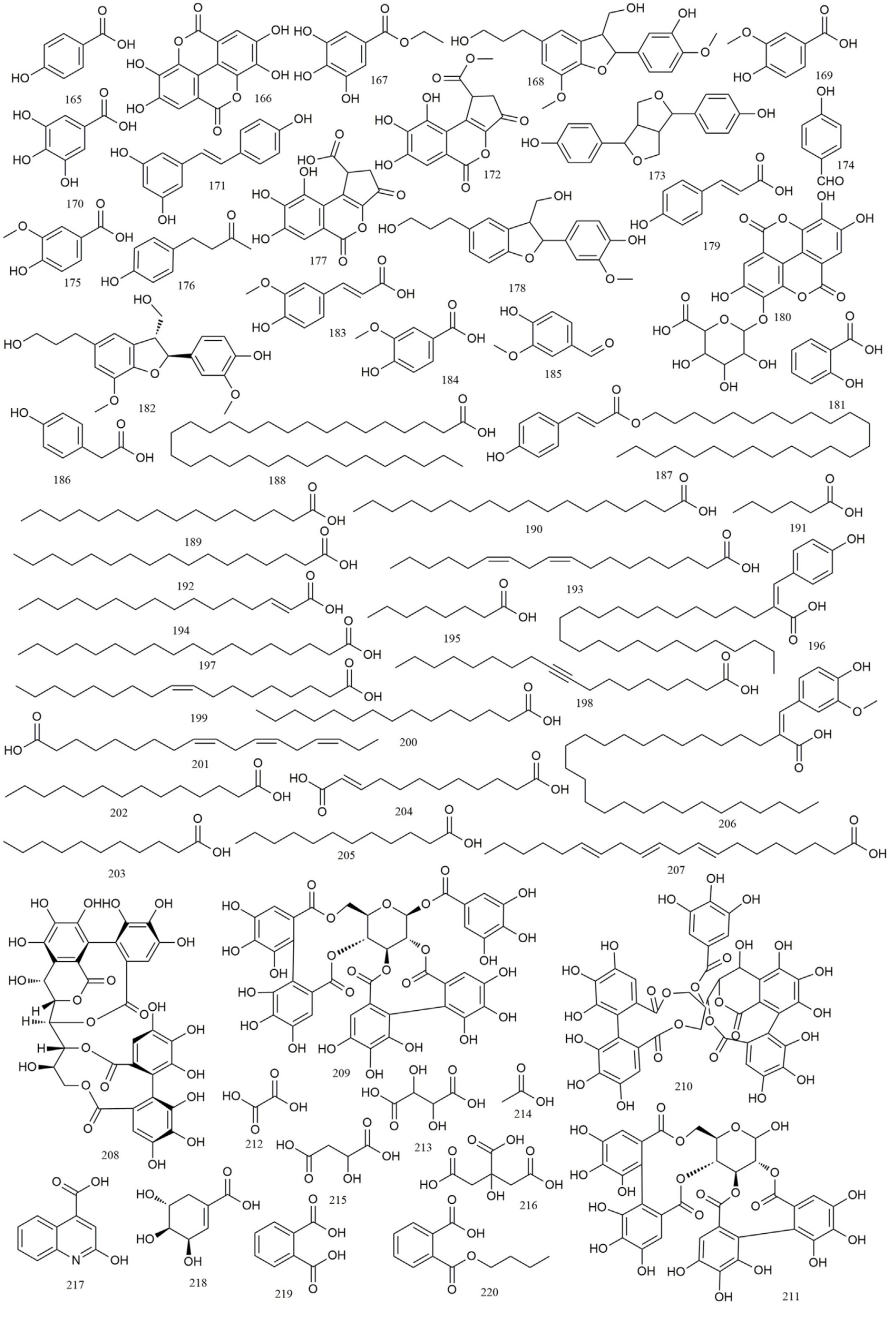
Chemical structures of organic acids **(165–220)** isolated from *R. chingii*.

Organic acids are a class of carboxyl-group-containing compounds that could be found in numerous plants worldwide. *R. chingii* extracts contain a high percentage of organic acids. A total of 56 organic acids, including 23 phenolic acids (**165–187**), 20 fatty acids (**188–207**), 4 tannins (**208–211**), and 9 other compounds (**212–220**) have been reported mainly from the fruits of *R. chingii* (Pi and Wu, 2003; Lim et al., 2004; Dian et al., 2005; Guo, 2005; Liu, 2005; Xie et al., 2005; Chai, 2008; Cheng, 2008; You, 2009; You et al., 2009; Xiao et al., 2011; Han et al., 2013; Sun et al., 2013a; Xie et al., 2013b; Du et al., 2014; Han et al., 2014; Liu et al., 2014; Zhang, 2014; Guo, 2015; Zhang and Jiang, 2015; Chai et al., 2016; Li et al., 2018). Detailed information of these organic acid compounds is shown in **Table 1** (**165–220**) and **Figure 8**.

### Other Compounds

**Figure 9:**
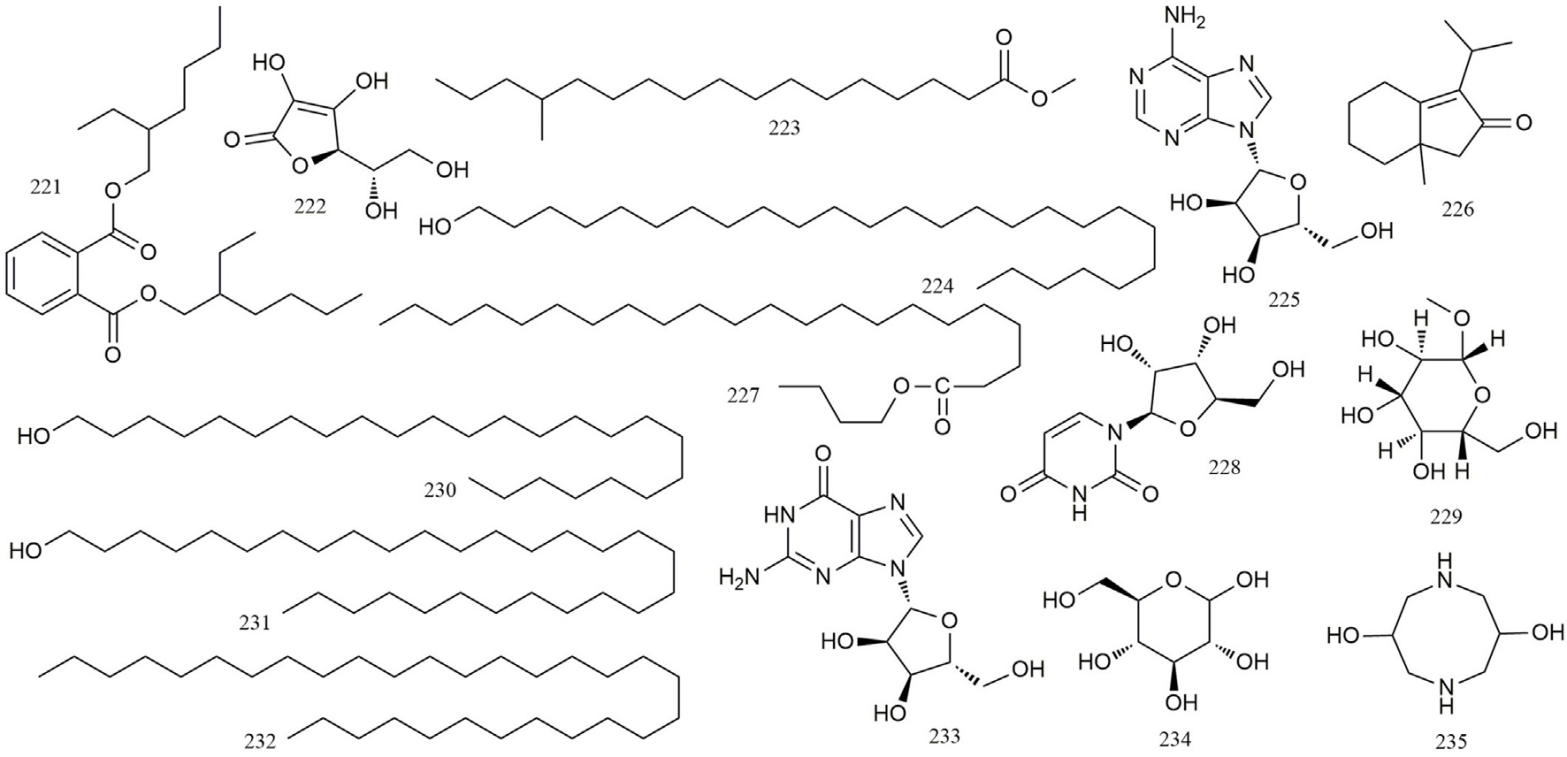
Chemical structures of other compounds **(221–235)** isolated from *R. chingii*.

In addition to these compounds mentioned above, a range of other compounds have also been isolated from *R. chingii*. Detailed information of these compounds is shown in **Table 1** (**221–235**) and **Figure 9** (Guo, 2005; Guo et al., 2007; Chai, 2008; Cheng, 2008; You, 2009; Kong et al., 2011; Xiao et al., 2011; Sun et al., 2013a; Xie et al., 2013b; Du et al., 2014; Zhang and Jiang, 2015).

## Pharmacological Activities of *R. chingii*

As a well-known medicinal plant in TCM, the fruits and leaves of *R. chingii* are widely used for the treatment of various diseases. The major pharmacological properties such as anticomplementary, anticancer, antioxidant, antimicrobial, anti-inflammatory, anti-hypotensive, anti-aging, antithrombotic, antidiabetic, neuroprotective, and anti-osteoporosis activities of this herbaceous medicine are summarized in **Table 2**, and the details will be further discussed below.

**Table 2:** Reported biological activities *in vitro* and *in vivo* of *R. chingii* crude extracts and fractions.

Extract	Reported activity	References
**ANTICOMPLEMENTARY ACTIVITY**		
Essential oils from fruits	Essential oils extracted by SE-ether had the best anti-complementary activity; at 0.2 mg/mL, its hemolysis inhibition exceeded 60% (*in vitro*).	Zhang and Jiang, 2015
Polysaccharides, flavonoids, saponins, and alkaloids from fruits	Flavonoids and saponins showed noteworthy anti-complementary activities; at 0.8 mg/mL, their hemolysis inhibition rates were 96.49% and 90.82%, respectively (*in vitro*).	Zhang et al., 2015a
**ANTICANCER ACTIVITY**		
Water extract from fruits	Inhibited matrix metalloproteinases-13 with an IC_50_ value of 0.04 µg/mL (*in vitro*).	Wang et al., 2011
Water extract from fruits	Anticancer potentials against human hepatoma SMMC-7721 cells with an IC_50_ value of 80 µg/mL (*in vitro*).	Hu, 2014
Essential oils from fruits	Essential oils extracted by SDE had the best anticancer activity against A549 cell lines with an inhibition rate of 58.13% at the concentration of 200 µg/mL (*in vitro*).	Zhang and Jiang, 2015
Polyphenolic composition from fruits	Anticancer potentials against human bladder cancer T24 cells. The IC_50_ values were 73.442 µg/mL, 55.294 µg/mL, and 26.686 µg/mL for 12 h, 24 h and 36 h, respectively (*in vitro*).	Li et al., 2018
Polysaccharides from fruits and leaves	Polysaccharides from leaves showed significant inhibitory activities on breast cancer cells MCF-7 proliferation; at 2 mg/mL its inhibition rate were 48.48 ± 0.55% and 66.30 ± 0.61% for 48 h and 72 h, respectively (*in vitro*).	Zhang et al., 2015b
Labdane-type diterpene glycosides from fruits	Compound 29 possessed remarkable cytotoxic activity against human lung cancer cells A549, with an IC_50_ value of 1.81 µg/mL (*in vitro*).	Zhong et al., 2015
Flavonoids and saponins from fruits	Anticancer potentials against human lung cancer cells A549. The inhibition rates were 65% and 62% (200 µg/mL), respectively (*in vitro*).	Zhang et al., 2015a
The ethyl acetate fraction from fruits	Antiproliferative potentials against HepG-2, Bel-7402, A549, and MCF-7 cancer cell lines (*in vitro*).	Zhang et al., 2017b
**ANTIMICROBIAL ACTIVITY**		
Flavonoids from fruits	Inhibited *Staphylococcus aureus*, *Bacillus subtilis*, *Escherichia coli*, and *Penicillium* with MIC values of 0.04 mg/mL, 0.08 mg/mL, 0.16 mg/mL, and 0.64 mg/mL, respectively (*in vitro*).	Zhu, 2012
70% ethanol extract from fruits	Inhibited fluconazole-resistant *Candida albicans* with a MIC_80_ value of 4.88-312.5 µg/mL.	Han et al., 2016
**ANTIOXIDANT ACTIVITY**		
Glycoprotein from fruits	*In vitro* antioxidant activity; *in vivo* promote the activities of CAT, SOD and GSH-PX.	Tian et al., 2010
Aqueous extract from fruits	Protected primary rat hepatocytes against (*t*-BHP)-induced rat hepatocytes by reversing cell viability loss, lactate dehydrogenase leakage and the associated glutathione depletion and lipid peroxidation (*in vitro*).	Yau et al., 2002
The ethyl acetate and *n*-butanol fractions from fruits	*In vitro* antioxidant activity (DPPH assay) with IC_50_ values of 3.4 and 4.0 µg/mL, respectively.	Ding, 2011
Flavonoids from fruits	*In vitro* antioxidant activity (DPPH assay and ABTS assay)	Zeng, 2015
Polysaccharides from fruits and leaves	*In vitro* antioxidant activity (DPPH assay). IE_50_ 754.33 µg/mL (F-Ps); 671.39 µg/mL (L-Ps).	Zhang et al., 2015b
Polyphenolic composition from fruits	*In vitro* antioxidant activity (DPPH assay) with an IC_50_ value of 33.912 µg/mL.	Li et al., 2018
95% ethanol extract from fruits	The ethyl acetate fraction and *n*-butanol fraction showed significant *in vitro* antioxidant activity (DPPH assay, reducing power assay and ORAC assay)	Zhang et al., 2017b
Flavonoids from fruits	The total flavonoids displayed the best *in vitro* antioxidant effect (DPPH assay, reducing power assay and ORAC assay), which was very close to ascorbic acid.	Zhang et al., 2015a
**ANTI-INFLAMMATORY ACTIVITY**		
Ethyl acetate fraction from fruits	Anti-inflammatory potentials against LPS-stimulated macrophage RAW264.7 cells (*in vitro*).	Zhang et al., 2015c
Polysaccharides from fruits and leaves	Anti-inflammatory potentials against LPS-stimulated murine macrophage RAW264.7 cells by decreasing NO production and increasing the TNF-α, iNOS and IL-6 gene expression (*in vitro*).	Zhang et al., 2015b
**ANTITHROMBOTIC ACTIVITY**		
70% ethanol fraction from leaves	Significant antithrombotic activity was observed in *in vitro* and *in vivo* tests.	Han et al., 2012
**NEUROPROTECTIVE ACTIVITY**		
80% ethanol extract from fruits	Significant improvements in learning and memory were observed, especially in rats receiving the chloroform and ethylacetate fractions (*in vivo*).	Huang et al., 2013
Different extracts from fruits	The high dose water extract (24 g/kg) was found to exhibit the best anti-amnesic effects on scopolamine and sodium nitrite (NaNO_2_)-induced amnestic models, while the crude drug showed the best anti-amnesic activity on 40% ethanol-induced amnestic models (*in vivo*).	Li et al., 2016a
Water extract from fruits	Ameliorated H_2_O_2_-induced damages of bEnd.3 cells (*in vitro*).	Liu, 2018
HYPOLIPIDEMIC ACTIVITY		
Water extract from leaves	Alleviated hyperlipidemia by decreasing TC and TG (*in vivo*).	Fan et al., 2007
**ANTIHYPOTENSIVE ACTIVITY**		
Ethanol extract from fruits	Induced the endothelium-dependent vasodilatory effect in rats *via* stimulation of the NO/guanylate cyclase/cGMP pathway and the Akt-eNOS pathway (*in vitro* and *in vivo*).	Su et al., 2014
**ANTI-AGING ACTIVITY**		
Glycoprotein from fruits	Anti-aging effect in mice by increasing the expression of anti-aging gene klotho and repairing the renal function (*in vivo*).	Zeng et al., 2018
**OTHER PHARMACOLOGICAL EFFECTS**		
Different extracts from fruits	*R. chingii* has mitogenic effects on spleen lymphocytes (*in vitro*).	Chen et al., 1995
Water extract from fruits	Regulated the hypothalamus-pituitary-sex gland axis (*in vivo*).	Chen et al., 1996
20% ethanol extract from fruits	Protected retinal ganglion cells from H_2_O_2_-induced cell death by increasing the Bcl-2 protein expression and decreasing Bax protein expression (*in vitro*).	Li, 2017

### Anticomplementary Activity

Several studies demonstrated that the extracts of *R. chingii* possess anticomplementary activity. Zhang and Jiang employed a complement fixation test to assess the *in vitro* anticomplementary activity of the essential oils from fruits of *R. chingii* by three different extraction methods [steam distillation extraction (SDE), soxhlet extraction (SE) with ethanol, and SE with ether]. The results showed that the essential oils obtained by SE-ether had the strongest anticomplementary effect, even stronger than heparin (control) (Zhang and Jiang, 2015). The flavonoids and saponins extracted from *R. chingii* also showed noteworthy anticomplementary activities when compared to its polysaccharides and alkaloids. The hemolysis inhibition rates of the flavonoids and saponins were 96.49% and 90.82% (at the concentration of 0.8 mg/ml), respectively, which were even higher than heparin sodium (Zhang et al., 2015a).

### Anticancer Activity

The antitumor effects of the various extracts of *R. chingii* have been extensively investigated through a large number of *in vivo* and *in vitro* experiments. Wang et al. (2011) found that the water extract of *R. chingii* could inhibit the activities of matrix metalloproteinases-13 with an IC_50_ value (half maximal inhibitory concentration) of 0.04 μg/ml. The results suggested that this herbal medicine may be used for the treatment of cancer. Another study showed that the water extract of *R. chingii* gave rise to a dose-dependent antiproliferative effect on hepatocellular carcinoma cells with an IC_50_ value of 80 μg/ml (Hu, 2014). Anticancer activity was also reported for the essential oils from the unripe fruits of *R. chingii* by *in vitro* MTT cytotoxicity assay against A549 cell lines. The results showed that the essential oils extracted by SDE exhibited stronger activity than SE-ethanol, which may be due to the extract obtained by SDE, which had a higher content of unsaturated fatty acids (Zhang and Jiang, 2015). An *in vitro* study showed that polyphenolic composition in the fruits of *R. chingii* could inhibit the proliferation and induce apoptosis of human bladder cancer T24 cells remarkably in a dose-dependent and time-response manner. The IC_50_ values were 73.442, 55.294, and 26.686 μg/ml for 12, 24, and 36 h, respectively (Li et al., 2018). In a similar study, Zhang et al. (2015b) evaluated the anticancer activity of the polysaccharides from *R. chingii via* MTT assay and found that inhibitory activities on breast cancer cells’ MCF-7 and liver cancer cells’ Bel-7402 proliferation were also concentration- and time-dependent. From 70% ethanol extract of the fruits of *R. chingii*, Zhong et al. (2015) isolated three new labdane-type diterpene glycosides and *in vitro* tests of these compounds for anticancer activity showed that compound 29 possessed remarkable cytotoxic activity against A549 (human lung cancer cell line), with an IC_50_ value of 1.81 μg/ml (2.32 μM). Furthermore, tiliroside, a representative flavonoid isolated from *R. chingii*, induced the apoptosis of A549 cells in a dose-dependent manner, with an IC_50_ value of 113.41 ± 1.89 μg/ml (190.76 ± 3.18 μM) (Zhang et al., 2015a). In 2017, Zhang et al. (2017b) investigated the antiproliferative ingredients in the fruits of *R. chingii* by using bio-assay guided isolation, and found that tormentic acid possessed notable cytotoxicity activities against HepG-2, Bel-7402, A549, and MCF-7 cancer cell lines with the IC_50_ values of 40.57, 54.22, 62.36, and 24.23 μg/ml, respectively. All these results described above suggest that *R. chingii* has an exact effect on prevention of cancer. However, a common mechanism about the exact cellular and molecular targets needs to be fully elucidated and the diversity of extracts makes data interpretation difficult.

### Antimicrobial Activity

Antimicrobial activity, an important effect of *R. chingii*, had been comprehensively studied. A moderate antibacterial activity was evident for the flavonoids from *R. chingii* against *Staphylococcus aureus*, *Bacillus subtilis*, *Escherichia coli*, and *Penicillium* with MIC (minimum inhibitory concentration) values of 0.04, 0.08, 0.16, and 0.64 mg/ml, respectively. However, it could not inhibit the growth of *Saccharomyces cerevisiae*, *Rhizopus*, and *Mucor* (Zhu, 2012). In addition, *R. chingii* extract combined with fluconazole displayed synergistic antifungal activity on fluconazole-resistant *Candida albicans* with an MIC_80_ (the lowest concentration to inhibit 80% of fungal growth) value of 0.0625–16 μg/ml for fluconazole and 4.88–312.5 μg/ml for the 70% ethanol extract of *R. chingii* (Han et al., 2016).

### Antioxidant Activity

Oxidative stress by free radicals is a significant event in the cell, which is associated with a wide range of human degenerative diseases (Bi et al., 2016). The glycoprotein from *R. chingii* showed significant *in vitro* antioxidant activity *via* free radical scavenging assay and reducing power assays. An in-depth *in vivo* study revealed that the glycoprotein could significantly increase the activities of catalase (CAT), superoxide dismutase (SOD), and glutathione peroxidase (GSH-P_X_) in serum, liver, and brain tissues of rats, which also confirmed the strong reducing power of the glycoprotein (Tian et al., 2010). The aqueous extract of *R. chingii* has also been reported to reverse *tert*-butyl hydroperoxide (*t*-BHP)-induced oxidative damage in rat hepatocytes by inhibiting lactate dehydrogenase leakage, lipid peroxidation, and the associated glutathione depletion (Yau et al., 2002). Moreover, among nine compounds isolated from the fruits of *R. chingii*, methyl (3-hydroxy-2-oxo-2,3-dihydroindol-3-yl)-acetate, vanillic acid, kaempferol, and tiliroside displayed antioxidative capacity. Their IC_50_ values were 45.2, 34.9, 78.5, and 13.7 μM, respectively (ascorbic acid, 131.8 μM) (Ding, 2011). Zeng et al. studied the *in vitro* antioxidant capacities of the total flavonoid contents of *R. chingii* by the 1,1-diphenyl-2-picrylhydrazyl (DPPH) and 2,2’-azino-bis 3-ethylbenzothiazoline-6-sulphonic acid (ABTS) methods. The results showed that the total flavonoid content exhibited a significant correlation with antioxidant activity in the DPPH assay (*r* ^2^ = 0.758, ρ = 0.004) and the ABTS assay (*r* ^2^ = 0.788, ρ = 0.002) (Zeng et al., 2015). Zhang et al. (2015b) studied the activities of polysaccharides from *R. chingii* fruit (F-Ps) and leaf (L-Ps) through DPPH scavenging assay and found that the scavenging activities of F-Ps and L-Ps had almost 10 folds lower antioxidant potential than the vitamin C with half inhibition effect (IE_50_) values of 754.33 and 671.39 μg/ml, respectively. Similarly, the polyphenolic composition in the fruits of *R. chingii* exhibited high DPPH scavenging effect with an IC_50_ value of 33.912 μg/ml, which was half of the standard ascorbic acid (Li et al., 2018). In 2017, an interesting study investigated the antioxidant effects of fruits of *R. chingii* by using the DPPH assay, reducing power assay and oxygen radical absorbance capacity (ORAC) assay, and the results revealed that the ethyl acetate fraction and *n*-butanol fraction were found to be the most potent (Zhang et al., 2017b). The polysaccharides, flavonoids, saponins, and alkaloids extracted from *R. chingii* were also assessed for their antioxidant activity through the same methods. The results indicated that total flavonoids displayed the best antioxidant effect, which was very close to ascorbic acid (Zhang et al., 2015a). From the results mentioned above, we can conclude that the strong antioxidant activity of *R. chingii* might be predominantly related to the presence of the glycoproteins and phenolic compounds, especially flavonoids. Additionally, it is worthy to note that the *in vitro* experiments used to test total antioxidant are not specific and prone to interferences, which may give unreliable results. Therefore, further *in vivo* studies are needed to validate these results.

### Anti-Inflammatory Activity

Sun et al. (2013b) extracted a new compound called goshonoside-G from the fruits of *R. chingii*. This compound possessed notable inhibitory effect on NO production in LPS-stimulated macrophage RAW264.7 cells with an IC_50_ value of 54.98 μg/ml. In bio-assay guided fractionation of the ethanol extract of *R. chingii*, which provided the best anti-inflammatory effect, tiliroside, astragalin, hyperoside, quercitrin, and kaempferol 3-rutinoside were isolated. Among the flavonoid glycosides, tiliroside possessed the strongest inhibitory effect on NO production in LPS-stimulated macrophage RAW 264.7 cells with the inhibitory rate of 30.4% at a concentration of 100 μg/ml, which was very close to that of dexamethasone at a concentration of 50 μg/ml. Western blot and RT-PCR showed that the underlying mechanism of the suppression of inflammatory reactions by tiliroside may be due to its modulation of a signaling mitogen-activated protein kinase (MAPK) and pro-inflammatory cytokines activities (Zhang et al., 2015c). In addition, the polysaccharides from leaves and fruits induced a dose-dependent (2–400 μg/ml) inhibition of the nitric oxide (NO) production in murine macrophage RAW 264.7 cells through suppressing the TNF-α, iNOS, and IL-6 gene expression (Zhang et al., 2015b). Therefore, flavonoid glycosides and polysaccharides along with goshonoside-G of the plant could be considered as potential anti-inflammatory agents.

### Antithrombotic Activity

The 70% ethanol fraction from an aqueous extract of *R. chingii* leaves was found to treat thrombosis through inhibiting the aggregation of blood platelets using activity tests carried out *in vitro* and *in vivo*. The bio-guided isolation of the extract yielded six compounds (salicylic acid, kaempferol, quercetin, tiliroside, quercetin 3-*O*-β-D-glucopyranoside, and kaempferol 3-*O*-β-D-glucopyranoside). Their anticoagulant activities were examined using plasma recalcification time (PRT) test. It is noteworthy that kaempferol, quercetin, and tiliroside obviously delayed PRT in blood at a concentration of 2 mg/ml, while salicylic acid, quercetin 3-*O*-β-D-glucopyranoside, and kaempferol 3-*O*-β-D-glucopyranoside demonstrated the weakest effect in the *in vitro* experiment (Han et al., 2012).

### Neuroprotective Activity

Huang et al. investigated whether or not *R. chingii* was involved in attenuating learning and memory deficits on a classical model of Kidney *Yang* Deficiency Syndrome (KDS-*Yang*) in Alzheimer’s disease rats induced by D-galactose combined with hydrocortisone. Morris water maze tests demonstrated significant improvements in learning and memory, especially in rats receiving the chloroform and ethylacetate fractions of *R. chingii* (Huang et al., 2013). The major mechanism may be that *R. chingii* could protect neurons in rat hippocampal CA1 region by increasing choline acyltransferase (ChAT) activity but decreasing acetylcholinesterase (AChE) activity and Tau protein expression. The possible memory-enhancing effects of different extracts of *R. chingii* on amnesic rats induced by scopolamine, sodium nitrite, and 40% ethanol were also studied by assessing a Morris water maze test. The results showed that the high-dose water extract (24 g/kg) exhibited the best anti-amnesic effects on scopolamine and sodium nitrite (NaNO_2_)-induced amnestic models, while the crude drug showed the best anti-amnesic activity on 40% ethanol-induced amnestic models (Li et al., 2016a). Moreover, Liu et al. (2018) demonstrated that the water extract of *R. chingii* could ameliorate H_2_O_2_-induced damages of brain microvascular endothelial cells (bEnd.3 cells) *via* regulating the expression of apoptosis-related proteins. In addition, two flavonoids (kaempferol and quercetin) isolated from *R. chingii* were investigated for neuroprotective activity. It was observed that at 80 μmol/L concentration, both compounds significantly inhibited the decrease of cell viability (MTT reduction), prevented membrane damage (LDH release), scavenged ROS formation, and attenuated the decrease of malondialdehyde (MDA) in H_2_O_2_-induced PC12 cells (Zhao et al., 2018). These abovementioned results of preclinical investigations show that *R. chingii* may be a promising herbal medicine to combat nerve injury.

### Antidiabetic Activity and Hypolipidemic Activity

Xie et al. reported antihyperglycemic effects of raspberry ketone in the alloxan-induced diabetic rat model, which were beneficial for the treatment of diabetes. The study showed that raspberry ketone reduced the level of the blood glucose, protected the normal physiological function of pancreatic β cells, and stimulated insulin secretion by effectively inhibiting the oxidative stress (Xie et al., 2012). Another study showed that raspberry ketone could significantly promote glucose uptake in HepG2 cells by increasing the IRS-1 protein expression and decreasing SHP-1 mRNA gene expression (Xie et al., 2014).

The hypolipidemic activity of the leaves from *R. chingii* was evaluated in the hyperlipidemia rats induced by a high-fat diet and adults with hyperlipidemia. The results revealed that treatment with raspberry leaves exhibited significant hypolipidemic effect, indicated by reduced level of serum total cholesterol (TC) and triacylglycerols (TGs). Therefore, it suggested that raspberry leaves could be further explored as a therapy for the treatment of hyperlipidemia diseases (Fan et al., 2007).

### Anti-Osteoporotic Activity

Liang et al. (2015) isolated a novel compound, rubusin B, and six known compounds from the fruits of *R. chingii*, and an *in vitro* study showed that rubusin B, kaempferol, rubusin A, and quercetin exhibited anti-osteoporotic activities with different characteristics. Quercetin and kaempferol had a direct stimulatory effect on alkaline phosphatase (ALP) activity and bone formation, while rubusin A and B could effectively attenuate osteoclastic resorption even at a very low concentration (0.01 ppm).

### Antihypotensive Activity

Recently, it was shown that the ethanol extract of *R. chingii* could induce the endothelium-dependent vasodilatory effect in rats, *via* stimulation of the NO/guanylate cyclase/cGMP pathway and the Akt-eNOS pathway (Su et al., 2014).

### Anti-Aging Activity

A novel glycoprotein isolated from *R. chingii* exhibited notable anti-aging effect in the D-galactose-induced aging mice model by increasing the expression of anti-aging gene klotho and repairing the renal function (Zeng et al., 2018).

### Other Pharmacological Effects

In addition to the bio-activities mentioned above, some other pharmacological effects of *R. chingii* and its constituents were also reported. Chen et al. (1995) demonstrated that *R. chingii* has mitogenic effects on spleen lymphocytes. They also found that *R. chingii* could regulate the hypothalamus–pituitary–sex gland axis (Chen et al., 1996). Li (2017) reported that *R. chingii* could protect retinal ganglion cells from H_2_O_2_-induced cell death by increasing the Bcl-2 protein expression and decreasing Bax protein expression.

## Toxicity

Limited data are available concerning the safety assessments of *R. chingii*. In an acute toxicity test, the dose of the water extract of *R. chingii* leaves used in mice was 20 g/kg/day, and it did not induce any toxicity sign or death in 2 weeks (Tang et al., 2007). The potential adverse effects of *R. chingii* leaves were also determined by a repeated dose oral toxicity study, which was conducted on Wistar rats administered for 90 days at oral dosages of 2.5, 5, and 10 g/kg. The researchers found no significant differences between groups in body weights, food consumption, blood biochemistry, organ weights, gross pathology, and histopathology. Further study indicated that *R. chingii* leaves had no mutagenic or genotoxic effect using the Ames test, bone marrow micronucleus test, and sperm aberration test (Tang et al., 2007). Based on the results described above, we can conclude that *R. chingii* leaves are not toxic and hence reliably safe for use for pharmacological purposes. However, more in-depth investigations are still needed to explore the toxicity of the fruits of *R. chingii* to human health.

## Quality Control

It is well known that the inherent quality of herb medicine may vary significantly in different geographical conditions and different harvest times (Zhang et al., 2018). In the Chinese Pharmacopoeia (2015), the contents of ellagic acid and kaempferol-3-*O*-rutinoside in *R. chingii* should not be less than 0.2% and 0.03%, respectively (Chinese Pharmacopoeia Commission, 2015). It is extensively accepted that the multiple components of TCM are responsible for their curative effects by exerting their synergistic effects on multiple targets and levels (Li et al., 2016b). Thus, relying only on the two components for quality control seems insufficient to determine the strengths and weaknesses of *R. chingii*. With the advancement of analytical tools, the multi-component determination has been extensively used for comprehensive quality assessment of *R. chingii*. A total of 21 compounds: tiliroside (Chai et al., 2009), kaempferol (Xie et al., 2015; Ping et al., 2016), gallic acid (Li and Tan, 2008), ellagic acid, quercetin-3-*O*-β-D-glucopyranoside, kaempferol-3-*O*-rutinoside, goshonoside-F5 (Han et al., 2013), rutin (Zhang et al., 2017a), hyperoside (Chen et al., 1996), astragalin (Zhong et al., 2014; Ma et al., 2017), quercetin (Cheng et al., 2012), maslinic acid, 2α-hydroxyursolic acid, oleanic acid (Cao et al., 2017), ursolic acid, arjunic acid, 2α,3α,19α-trihydroxy-12-oleanen-28-oic acid, euscaphic acid (Guo et al., 2005), adenosine, brevifolin carboxylic acid, and ethyl gallate (Chai et al., 2016), have been quantified by HPLC or CE by different research groups (Chen et al., 2006). The volatile constituents such as hexadecanoic acid, tetradecanoic acid, and acetic acid were detected by GC/MS (Han et al., 2014; Zhang and Jiang, 2015). In addition, a pharmacokinetic study was carried out to determine quercetin-3-*O*-β-D-glucopyranoside, kaempferol-3-*O*-rutinoside, and tiliroside in rat plasma after oral administration of *R. chingii* to rats (Zan et al., 2018). However, there is still no unified method for quality control and fingerprinting of *R. chingii*. The quantitative analysis of *R. chingii* is listed in **Table 3**.

**Table 3:** Quantitative analysis for the quality control of *R. chingii*.

Analytes	Method	Results	References
Tiliroside	HPLC	0.0700% to 0.0338% (contents).	Chai et al., 2009
Tiliroside, Kaempferol	HPLC	0.1769–0.5150 mg/g and 6.7–23.9 µg/g, respectively (contents).	Ping et al., 2016
Gallic acid	HPLC	5.24–104.8 µg/ml (linear range); 97.6% (average recovery).	Li and Tan, 2008
Ellagic acid, Quercetin-3-*O*-β-D-glucopyranoside, Kaempferol-3-*O*-rutinoside, Tiliroside, Kaempferol, Goshonoside-F5	HPLC-UV, HPLC-ELSD	0.078%–0.315%, 0.001%–0.015%, 0.006%–0.065%, 0.003%–0.046%, 0.001%–0.003%, 0%–0.127%, respectively (contents).	He et al., 2013
Ellagic acid, Rutin, Hyperoside, Quercetin-3-*O*-β-D- glucopyranoside, Kaempferol-3-*O*-rutinoside, Tiliroside	HPLC	0.0610%–0.4333%, 0.0008%–0.0024%, 0.0010%–0.0050%, 0.0011%–0.0077%, 0.0058%–0.0284%, 0.0231%–0.1025%, respectively (contents).	Zhang et al., 2017a
Astragalin, Tiliroside, Quercetin, Kaempferol	HPLC	38.24–91.04, 208.14–488.80, 205.68–1624.06, 22.44–84.72 µg/g, respectively (contents).	Ma et al., 2017
Kaempferol-3-*O*-rutinoside, Astragalin	HPLC	0.011–0.080 and 0.005–0.020 mg/g, respectively (contents).	Zhong et al., 2014
Rutin, Tiliroside, Quercetin	UPLC	0.0097–0.0500, 0.21–0.73, and 0.023–0.061 mg/g, respectively (contents).	Cheng et al., 2012
Maslinic acid, 2α-Hydroxyursolic acid, Oleanic acid	HPLC	0.032%–0.075%, 0.009%–0.053%, and 0.072%–2.087%, respectively (contents).	Cao et al., 2017
Kaempferol	HPLC	19.91 to 22.26 µg/g (contents).	Xie et al., 2015
Fingerprint	HPLC	A total of 15 common peaks were found in the HPLC fingerprints of *R. chingii*.	Chen et al., 2006
Oleanolic acid, Ursolic acid, Maslinic acid, 2α-Hydroxyursolic acid, Arjunic acid, 2α,3α,19α-Trihydroxy-12- Oleanen-28-oic acid, Euscaphic acid	CE (Capillary electrophoresis)	This method is rapid, precise, and reproducible, and is useful for quantitative analysis of the triterpenes	Guo et al., 2005
Volatile constituents	GC/MS	A total of 37 constituents were identified from the leaves of *R. chingii*, mainly including hexadecanoic acid (44.97%), tetradecanoic acid (10.88%), and acetic acid (4.13%).	Han et al., 2014
Adenosine, Gallic acid, Brevifolin carboxylic acid, Ethyl gallate, Ellagic acid, Kaempferol-3-*O*-rutinoside, Astragalin, Tiliroside	UPLC	The contents of the eight components vary significantly in the fruits of *R. chingii* collected from different habitats. And only two compounds, namely, adenosine and ellagic acid, are determined in the ripe fruits of *R. chingii*.	Chai et al., 2016
Volatile constituents	GC/MS	A total of 58 volatile compounds were identified from the unripe fruits of *R. chingii*.	Zhang and Jiang, 2015

## Conclusion and Future Perspectives

*R. chingii* is a nutritive plant commonly used as a functional food and medicine in China. It has been applied in clinical practice successfully for centuries to tonify the kidney, control nocturnal emissions, and reduce urination (Han et al., 2012). Although chemical compositions and biological activities of this medical plant are well documented, more conclusive studies are still needed to fill certain specific gaps in *R. chingii* science.

Firstly, and particularly, it is noteworthy that most pharmacological studies on *R. chingii* have only been conducted in animal models, cell models, and other *in vitro* experiments. Therefore, comprehensive placebo-controlled and double-blind clinical trials should be undertaken in the future to provide remarkable evidence for these positive findings on the efficacy of *R. chingii*. Besides, some of the pharmacological studies were carried out at too high doses that could hardly be translated to clinical practice and more in-depth investigations are needed to standardize the best dosage for these claimed bioactivities of *R. chingii* in ethnomedicine. In addition, the exact mechanisms of many medicinal properties of this herb still remain vague to date; thus, additional studies to better identify the functions and molecular targets seem to be necessary.

Secondly, most pharmacological activities were measured using uncharacterized crude extracts of *R. chingii*, and this makes it hard to reproduce the results of these investigations and elucidate the link between activity and particular compounds. Additionally, most of these phytochemicals were isolated from the fruits, and the chemical composition of other parts of this plant was largely unknown. Therefore, in-depth phytochemical investigations of all parts of *R. chingii* based on bio-guided isolation strategies are still needed, which may lead to the expansion of existing therapeutic potential of this miracle herb.

Thirdly, toxicological studies are important to understand the safety profile of herbal drugs, but data on toxicological aspects of *R. chingii* remain unexplored. The only toxicological study about *R. chingii* was conducted in the leaf extract, which revealed its non-toxic nature. Hence, to ensure a full utilization of the medicinal resource, further relative systematic toxicity and safety evaluation studies were quite considerable and necessary, especially in fruit extract and other effective extracts, to meet the Western standards of evidence-based medicine.

Fourthly, pharmacokinetic studies involving *R. chingii* are very limited and only focus on a few biological active substances present in *R. chingii*, which do not fully reflect the pharmacokinetic properties of this herb medicine. Thus, further investigations should be carried out to assess the absorption, distribution, metabolism, and excretion of the crude extracts of this plant *in vivo*. Additionally, metabolic studies of single isolated compounds in *R. chingii* should be strengthened, which could provide a scientific basis for clarifying the major metabolic route and action mechanism and defining the bio-active components responsible for the curative effects. Meanwhile, the identification of unknown metabolites may contribute to the drug discovery and development process.

Lastly, and importantly, because of the complex composition of TCM, quality control of TCM is a great challenge and has become a key factor to restrict its modernization process. Thus, setting up an effective and standardized quality control method of *R. chingii* is indispensable and emergent, which is crucial for ensuring the safety and efficacy of this medicinal product. In addition, good plant practice ought to be enforced to fulfill quantity and quality requirements for *R. chingii*.

## Author Contributions

GY and ZL searched the literature, collected the data, and drafted the manuscript. GY and WW contributed to analysis and manuscript preparation. YL and YZ helped check the chemical structures and formula. YS provided comments on the manuscript. All authors read and approved the final manuscript.

## Funding

This study was supported by the Start-up fund from Beijing University of Chinese Medicine to YS (No. 1000061020044 and No. 1000041510052).

## Conflict of Interest Statement

The authors declare that the research was conducted in the absence of any commercial or financial relationships that could be construed as a potential conflict of interest.
